# The distributional impact of a green payment policy for organic fruit

**DOI:** 10.1371/journal.pone.0211199

**Published:** 2019-02-07

**Authors:** Erik Nelson, John Fitzgerald, Nathan Tefft

**Affiliations:** 1 Department of Economics, Bowdoin College, Brunswick, Maine, United States of America; 2 Department of Economics, Bates College, Lewiston, Maine, United States of America; Universitat Jaume I, SPAIN

## Abstract

Consumer spending on organic food products has grown rapidly. Some claim that organics have ecological, equity, and health advantages over conventional food and therefore should be subsidized. Here we explore the distributive impacts of an organic fruit subsidy that reduces the retail price of organic fruit in the US by 10 percent. We estimate the impact of the subsidy on organic fruit demand in a representative poor, middle income, and rich US household using three analytical methods; including two econometric and one machine learning. We do not find strong evidence of regressive redistribution due to our simulated organic fruit subsidy; the poor household’s relative reaction to the subsidy is not much different than the reaction at the other two households. However, the infra-marginal savings from the subsidy tend to be larger in richer households.

## Introduction

One could argue that organic food production is a Clean Technology (CT) that deserves subsidization. CT produces a good with fewer environmental and human health impacts than a similar good produced by a more mature but ‘dirtier’ technology [1]. However, the goods produced by a CT have higher average production costs than the very similar goods produced by the ‘dirtier’ technology [1]. Despite incentivizing industry to incur higher-than-necessary production costs, subsidization of CT can make society better off if its use incidentally generates benefits that are greater than additional costs (e.g., [2]). Not only can subsidization of a CT generate immediate social welfare if the total subsidy is less than the external benefit created by use of the CT, but subsidization of the CT’s use accelerates the decline in the clean technology’s average cost of production via learning-by-doing (e.g., [3–4]) and the creation of economies of scale (e.g., [5]), promising even more welfare in the future.

Like other CT, organic agriculture produces goods similar to goods produced by ‘dirtier’ technology (conventional or “industrial” agriculture), but operates at a higher average cost ([6–9], S1 Supporting information) and, in many cases, generates greater uncertainty in producer returns [7,10–13]. However, like the impact of adopting other CT, organic systems can generate several incidental social, human health, and environmental benefits relative to production and consumption with the conventional technology. For example, studies have found that landscapes with more organic farms also have more equitable and vibrant local economies [14]. Organic farms in the US tend to be smaller and less capitalized, and therefore are a countervailing force against the overall trend of farm consolidation across the rural US. In 2014, 12,595 farms that covered 3,642,933 acres were certified organic, meaning an average farm size of 289 acres. In contrast, there were 2,085,000 farms of all types in the US in 2014, with an average size of 438 acres [15]. Further, the limited use of pesticides in organic farming has been found to significantly reduces illnesses and injuries among agricultural workers relative to conventional farming practices [16].

Some of the other human health attributed to organic consumption and organic production may or may not exist. For example, the organic process is perceived by many to produce food that is more nutritious and less likely to expose consumers to higher levels of dangerous chemicals relative to conventionally produced analogs. Whether these claims are true or not is hotly contested [17–26]. Regardless of the actual gains to private health from consuming organic foods, the *perception* of such gains mean that organic food persists as a *stated* preference for many households [16,27–28]. However, organic food price premiums mean these investments in (perceived) private health are less available to the households with limited food budgets [29]. Therefore, subsidizing the production or consumption of organic food would, theoretically, give poorer households greater capacity to access (perceived) health benefits.

Likewise, a positive environmental impact from organic production may be more perceived than real. On the positive side of the environmental impact ledger, organic production tends to have lower irrigation requirements, leads to less soil carbon loss [30], uses less energy [31–32], and supports more biodiversity [33–34] than conventional production. On the negative side of the environmental impact ledger, organic production’s reliance on manure for nitrogen instead of synthetic fertilizers means lower yields [32] and lower plant uptake of nitrogen relative to conventional farming (synthetic fertilizers are designed to make their nitrogen available when needed by the plant but manure is not; see [35]). Therefore, as demand for organics increase, landscapes dominated by organic production experience 1) greater rates of land conversion, and therefore, greater losses in the ecosystem services produced by less-intensively used land, to compensate for lower organic yields and 2) greater eutrophication and acidification potential relative to landscapes dominated by conventional production [32]. A recent review of the organic production literature found that organic production’s indirect land use impact is so severe that it turns a process that is less polluting than conventional farming on a per unit of land basis into a more polluting process on a per unit of output basis [36]. Therefore, while popular opinion holds that organic production is better for the environment than conventional production [37–38], whether the adoption of organic farming generates net environmental benefit is an open question.

The real and perceived external benefits created by organic food production and consumption has led the European Union to subsidize organic food production. Dimitri and Oberholtzer [39] stress the economic efficiency gains that European policy-makers believe the policy induces:

“European governments support organic agriculture through green payments…for converting to and continuing organic farming. The economic rationale for these subsidies is that organic production provides benefits that accrue to society and that farmers lack incentives to consider social benefits when making production decisions. In such cases, payments can more closely align each farmer’s private costs and benefits with societal costs and benefits.”

While previous US farm bills have provided some one-time subsidies to US farmers switching from conventional to organic farming [11], this external support has been small relative to European support for organic production. Therefore, the American Public Health Association, among others, has advocated adding an European-style green payment policy to the US farm bill [40] to better align US farmer’s private costs and benefits with societal costs and (perceived) benefits.

While the drive for an organic subsidy in the US seems to be largely motivated by economic efficiency concerns, in this paper we consider how the subsidy’s consumer benefits would be distributed across the US household income spectrum, about which there is little current evidence. CT subsidization policies tend to regressively re-distribute consumer welfare [41–45]. Therefore, assessing whether organic production subsidization would regressively redistribute US consumer welfare is highly relevant to organic food policy discussions, especially those that concern subsidization.

Richer households could benefit more from organic food subsidization if poorer households have less access to organic produce [46], if poorer households have *relatively* weaker demand for organic food [47], poorer households feel they cannot risk their limited food budget on perishable goods despite a price reduction [48], poorer households are less aware of shrinking organic price premiums caused by subsidization [29], or the subsidy is paid for by taxes that disproportionally affect the poor.

Here we explore the distributive impacts of an organic food subsidy—whether it is a production or consumption subsidy—that reduces the retail prices of organic fruit in the US by 10 percent from their 2011–2013 levels. First, we estimate the demand for organic fruit across US households stratified by annual income. Second, we use these estimated models to predict the impact of the subsidy on representative households’ 1) inframarginal gains from organic fruit consumption and 2) the change in organic fruit consumption. If the relatively affluent experience greater inframarginal gains *and* react more strongly to the lower organic food prices created by subsidization than less well-off households then the subsidy program would re-distribute even more welfare to the affluent. We focus on organic fruit because 1) it was widely available in all Americans markets in 2011–2013, our analytical timeframe, [49] and 2) organic produce, especially fruit, is the most popular organic food type [50].

*A priori* we suspected a broad subsidy of US organic fruit would primarily re-distribute surplus to wealthier US households. Previous research has found that urban households with more educated, older and married heads of house with at least one child at home, and *higher incomes* are more likely to buy organic produce, all else equal [27–28,51–56]. Therefore, if broad organic fruit subsidization were to reinforce these trends then subsidy-related costs could be incurred by many taxpayers to primarily improve the welfare of more educated and wealthier US households. Alternative impacts include organic fruit subsidies generating an equal bump in organic fruit consumption across US household types or, if the marginal utility from organic food consumption declines quickly, generating a relatively larger bump in organic fruit consumption for those with more modest means.

## Materials and methods

### Data

Our study is based on a sample of US household organic and conventional fruit purchases from 2011 through 2013. The data comes from the Nielsen Corporation’s Consumer Panel Data. Each year Nielsen recruits approximately 60,000 US households to record *each* purchase, food-related or not, they make over the course of the year. Sampled household purchase data, as well as the related household demographic information, can be projected to market, regional, and national levels using projection factors assigned to each household [57–58].

Using this dataset we generate the following data for each sampled household by month (household-month) from 2011 through 2013: 1) the ounces of type × variety fruit *i* that household *k* bought in month *m*, given by *o*_*ikm*_, where type is either organic or conventional and variety refers to apple, orange, strawberry, etc.; 2) household *k*’s expenditure on type × variety fruit *i* in month *m*, given by *e*_*ikm*_; 3) the average price of type × variety fruit *i* that household *k* faced in month *m*, given by *p*_*ikm*_ (in some cases we had to impute price; see S2 Supporting information); 4) several vectors of household *k*’s characteristics in month *m*, given by **X**_*km*_ and **C**_*km*_; and 5) household *k*’s status as a low (130% or less of their federal poverty line (FPL)), middle (130% to 500% of their FPL), or high (greater than 500% of their FPL) income household in month *m* (S3 Supporting information). The FPL varies by year and family size. For example, in 2011 the FPL was $22,350 for a family of four and $26,170 for a family of five. However, in 2013 the FPL was $23,550 for a family of four and $27,570 for a family of five. We coded households at 130% of their FPL or lower as low income because they are eligible for the Supplemental Nutrition Assistance Program (https://www.cbpp.org/research/a-quick-guide-to-snap-eligibility-and-benefits). We coded households at 500% or greater of their FPL as high income because many federal and nonprofit assistance programs (e.g., AIDS Drug Assistance Program, the Leukemia and Lymphoma Society’s financial assistance programs) are not available to households in this category while it is for all other households.

The vector **X**_*km*_ contains the household variables that previous research has flagged as affecting propensity to buy and overall consumption of organic produce, including the household’s monthly real income (December, 2013 $), household size, whether or not the household contains one or more children under 18, whether at least one head of household has a college degree, whether the household is headed by a married couple, and the race of the head of household. The vector **C**_*km*_ indicates *m*’s season × year (e.g., winter 2011, spring 2011, etc.), whether or not household *k* lives in a metro or non-metro county in month *m*, and which Nielsen Scantrack market household *k* lives in during month *m*. For estimation purposes, we also define **c**_*km*_⊂**C**_*km*_ where **c**_*km*_ only includes the season × year interaction dummy variables. All values in **X**_*km*_ and **C**_*km*_ stay fixed within a calendar year. See Table 1 for a summary of some of these data across sampled households.

**Table 1:** Summary of dataset by household income class and year. This table only includes data on fruit purchased with a Universal Product Code (UPC). Standard errors are in parentheses. Dollar values are in December, 2013 dollars. Data are not projected.

Household Income Class	Poor			Middle			Rich		
Year	2011	2012	2013	2011	2012	2013	2011	2012	2013
**Household-months (hhm)**	80052	78072	62904	418260	405048	357240	246792	243336	228012
**Mean organic expenditures / hhm**	0.06 (0.003)	0.07 (0.004)	0.10 (0.005)	0.09 (0.002)	0.10 (0.002)	0.14 (0.002)	0.17 (0.003)	0.20 (0.003)	0.28 (0.004)
**Mean conventional expenditures / hhm**	2.74 (0.02)	2.85 (0.02)	3.61 (0.03)	4.03 (0.01)	4.16 (0.01)	4.83 (0.01)	5.52 (0.02)	5.71 (0.02)	6.46 (0.02)
**Mean household size**	2.44 (0.006)	2.44 (0.006)	2.49 (0.006)	2.39 (0.002)	2.41 (0.002)	2.43 (.002)	2.26 (0.002)	2.29 (0.002)	2.31 (0.002)
**Fraction of urban hhm**	0.747	0.761	0.774	0.813	0.814	0.830	0.901	0.897	0.912

We include the market and metro classification variables in **c**_*km*_ and **C**_*km*_ to control for coarse differences in organic fruit availability across the US landscape [59]. We do not have the data to control for organic fruit availability at a fine scale. For example, grocery stores in higher income areas have been shown to offer healthier items [47] and more organic options [46] than those in poorer areas. Obviously, these fine-grain patterns of organic fruit availability could affect demand for organic fruit. However, some researchers have downplayed the importance of healthy food supply in a household’s neighborhood on determining demand for produce. For example, Allcott et al. [47] found that the entrance of a supermarket with healthy food options into a “food desert” does little to affect the food choices of neighborhood residents. The implication is that households will travel where necessary to within a larger market area to satisfy their preferences.

We only used fruit purchases, organic and conventional, recorded in the 2011–2013 Consumer Panel datasets that involved the scanning of a Universal Product Code (UPC) at a store. A fruit purchase was coded as organic if its UPC or item description indicated the item had the US Department of Agriculture (USDA) organic label. Therefore, if the description of a purchased product included a claim of organic but the item did not have the USDA organic label it was coded as conventional. In some cases, fruit purchases made with a UPC were recorded on a per item basis rather than a weight basis (e.g., two apples bought for $2 instead of 30 ounces bought for $2). Because our subsidy simulations manipulate organic fruit on a per weight basis (e.g., dollars per ounce) we converted all item-based expenditures and prices to ounces of expenditures and per ounce prices (see S1 Table for assumed fruit weights).

As of 2014, approximately 92 percent of organic food was sold via stores [60] and 40 percent of all fresh produce bought at US stores is done so with a UPC [47]. Therefore, given that UPC purchases represent a significant portion of all fruit purchases, our analysis of expected changes in UPC-coded fruit purchases due to a subsidy can plausibly be extended to cover all purchased fruit.

Finally, our dataset only includes 10 type × variety fruit combinations, including two ‘other’ categories. Specifically, *i* indexes organic varieties of apples, blueberries, oranges, strawberries, and “all other fruit” and conventional varieties of apples, blueberries, oranges, and strawberries, and “all other fruit” (S4 Supporting information). We focus on organic apples, blueberries, oranges, and strawberries because, based on expenditures, they are 4 of the 6 most popular organic fruit varieties in the US (S2 Table).

### Trends in the organic and conventional fruit expenditure and price data during 2011–2013

Using each household’s projection factors we found that total US household expenditures on organic fruits with a PUC increased from $144.82 million in 2011 to $211.53 million in 2013 (December, 2013 $) or 46.1% (all statistics and trends discussed in this section only refer to fruit, both organic and conventional, purchased with UPCs). Over the same time period, total US household expenditures on conventional fruits increased by 6.9% (Table 2). Overall, poor US households increased their expenditures on organic fruits between 2011 and 2013 by 68.7%, compared to the middle and high-income class’ 34.5% and 51.6% respective increases. However, on a per household basis, the high-income bracket not only bought more organic fruit than typical low income and middle-class households, they also experienced the greatest growth in organic fruit purchases between 2011 and 2013 (Table 2).

**Table 2:** Real US expenditures on fruit by household income class and year (December, 2013 $). This table only includes data on fruit purchased with a Universal Product Code (UPC). Household projection factors are used to extrapolate panel totals to national totals. See S3 Supporting information for details on income classification.

	Household Income Class	2011		2012		2013	
		Organic	Conventional	Organic	Conventional	Organic	Conventional
**Total Expenditures (Millions of $)**	**Low**	9.97	571.76	12.38	636.23	16.82	692.64
	**Middle**	57.16	2392.18	65.68	2621.65	76.88	2633.01
	**High**	77.70	2504.07	89.93	2375.93	117.83	2519.55
	**All**	144.82	5468.00	167.99	5633.82	211.53	5845.20
**Expenditures / Household**	**Low**	0.65	37.06	0.77	39.67	0.98	40.32
	**Middle**	1.16	48.59	1.27	50.70	1.53	52.40
	**High**	2.08	66.99	2.66	70.16	3.38	72.26
	**All**	1.42	53.59	1.65	55.45	2.07	57.14

The 2011 to 2013 growth in US household-level organic fruit purchases occurred at both the extensive and intensive margins. Growth on the extensive margin occurred across all three income brackets (Table 3). Among all US households, the number of households that only bought organic fruit in a given year and the number of households that bought some organic fruit in a given year increased by 80.0% and 35.7%, respectively, between 2011 and 2013. Conversely, over this same time period, the number of US households that only purchased conventional fruit fell 4.5%. The growth in “organic fruit-only” or “both varieties of fruit” households between 2011 and 2013 was greatest in the middle- income bracket (Table 3). As to the intensive margin, of the US households that were represented in the 2011 and the 2013 Consumer Panels, their total expenditures on organic fruit was 47.1% higher in 2013 than in 2011.

**Table 3:** Number of US households that bought organic, conventional fruit, or both in a calendar year by household income class and year (Millions of HHs). This table only includes data on fruit purchased with a Universal Product Code (UPC). Projection factors are used to extrapolate panel level results to national estimates. See S3 Supporting information for details on income classification.

	2011			2012			2013		
Household Income Class	Organic Only	Conventional Only	Both	Organic Only	Conventional Only	Both	Organic Only	Conventional Only	Both
**Low**	0.02	14.34	1.06	0.00	14.84	1.19	0.04	15.48	1.65
**Middle**	0.06	44.39	4.78	0.10	45.84	5.76	0.15	43.35	6.75
**High**	0.06	31.52	5.79	0.06	27.51	6.30	0.08	27.39	7.40
**All US**	0.15	90.25	11.64	0.16	88.20	13.25	0.27	86.22	15.80

**Fig 1:**
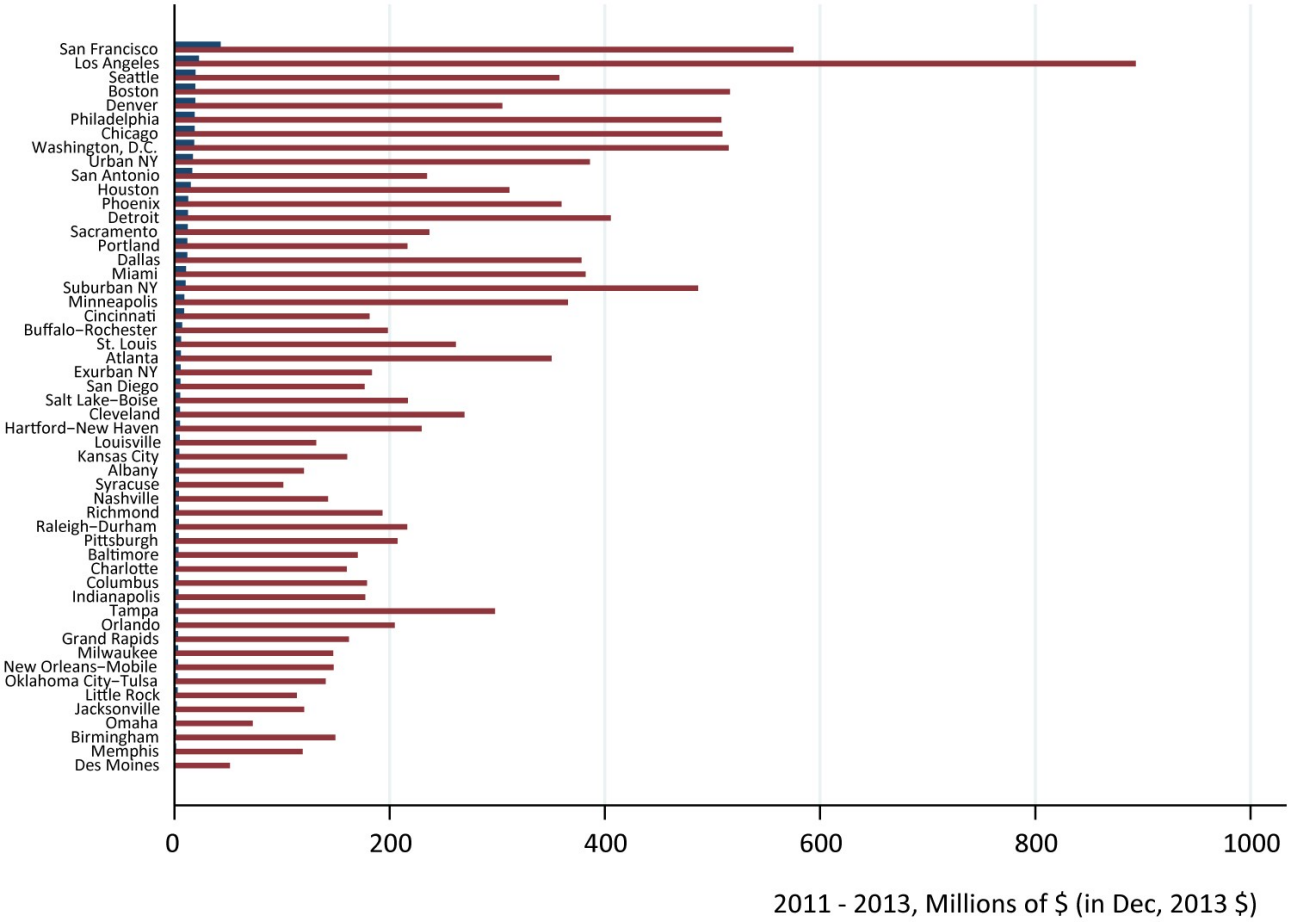
Conventional (red or gray bars) and organic (blue or dark bars) fruit expenditures by Nielsen Scantrack market, 2011 through 2013 (December, 2013 $). The y-axis is ordered in descending order by level of expenditures on organic fruit. This figure only includes fruit purchased with a Universal Product Code (UPC). All household expenditures in year *t* are inflated by households’ year *t* projection factor to arrive at market totals. A household’s year *t* projection factor indicates how many other households in that market the household in question represents in year *t*.

The spatial concentration of organic fruit purchases made between 2011 and 2013 was more intense than that of conventional fruit purchases. Using household projection factors, we found that consumers in the six (eleven) of the 52 Nielsen Scantrack markets that spent the most on organic fruit were responsible for a third (a half) of all organic fruit purchases (Fig 1). Conversely, households in the top eight (fourteen) markets for *conventional* fruit purchases made during the 2011 to 2013 period were responsible for a third (a half) of all conventional fruit purchases.

**Fig 2:**
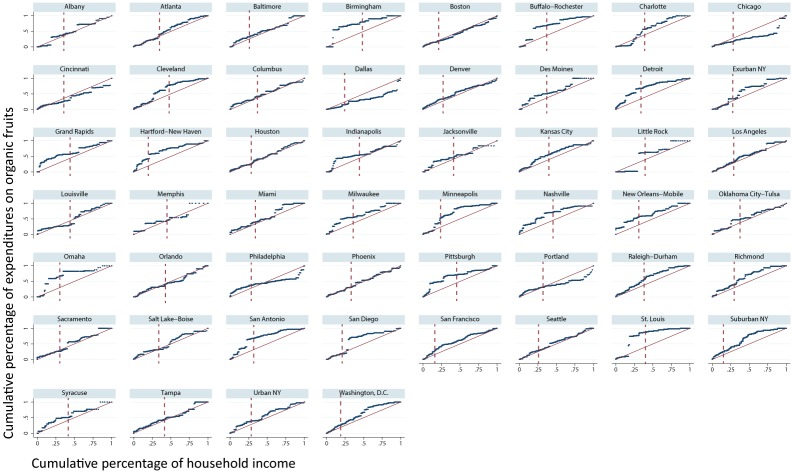
Lorenz curves of organic fruit expenditures across two-person households by Nielsen Scantrack, 2011 through 2013 (December, 2013 $). The dark line in each plot is the actual cumulative household income-household expenditure curve and the lighter line is the 45-degree line. The dashed vertical lines indicate the approximate break point between the middle-class and rich income categories along a market’s annual household income spectrum on the x-axis. Not included on the figure are the breakpoints between a market’s poor and middle-class households, which occur somewhere between 0.6 and 4.5 percent of cumulative income. All household expenditures in year *t* are inflated by households’ year *t* projection factor to arrive at market totals. This figure only includes fruit purchased with a Universal Product Code (UPC).

The pattern of 2011–2013 organic fruit purchases across households ordered by income *within* Scantrack markets was also often skewed. In Fig 2 and S1 Fig we display the cumulative proportion of a market’s expenditures on organic fruit from 2011–2013 against cumulative household income in each market. The figures also indicate the approximate break point between the middle-class and rich income categories along a market’s household income spectrum. We conduct this Lorenz Curve analyses for 2-person households (the most frequent household size type) (Fig 2) and for 3-person households (the most frequent household size that typically includes a child) (S1 Fig). In both 2-person and 3-person households, disproportionate gains in organic expenditures within a market, if they took place at all, *generally* took place somewhere in the middle or rich portion of the market’s income distribution. (In comparison, in almost every market, the poor and middle-class spend disproportionally more of their income on *conventional* fruit relative to richer households; see S2 and S3 Figs) There are some exceptions to this general rule. For example, poorer two-person households in Detroit and Nashville and poorer three-person households in Boston, Los Angeles, and Seattle purchased more than their fair share of organic fruit. Further, some markets had remarkably even distributions of organic fruit consumption across their income spectrums. This trend was especially evident in two-person households. Examples of markets with this trend include Boston, Columbus, Denver, Houston, Seattle, Orlando, and Phoenix.

Finally, we present two summaries of 2011–2013 organic fruit prices. At the national level, the annual average price series for each organic fruit is either monotonically increasing or does not display a trend across the years 2011 to 2013 (S3 Table). The trend in regional organic strawberry prices is uniform: in all regions the average annual organic strawberry price fell between 2011 and 2012 but then increased between 2012 and 2013. The other three organic fruits do not display such uniformity in regional price trends.

**Fig 3:**
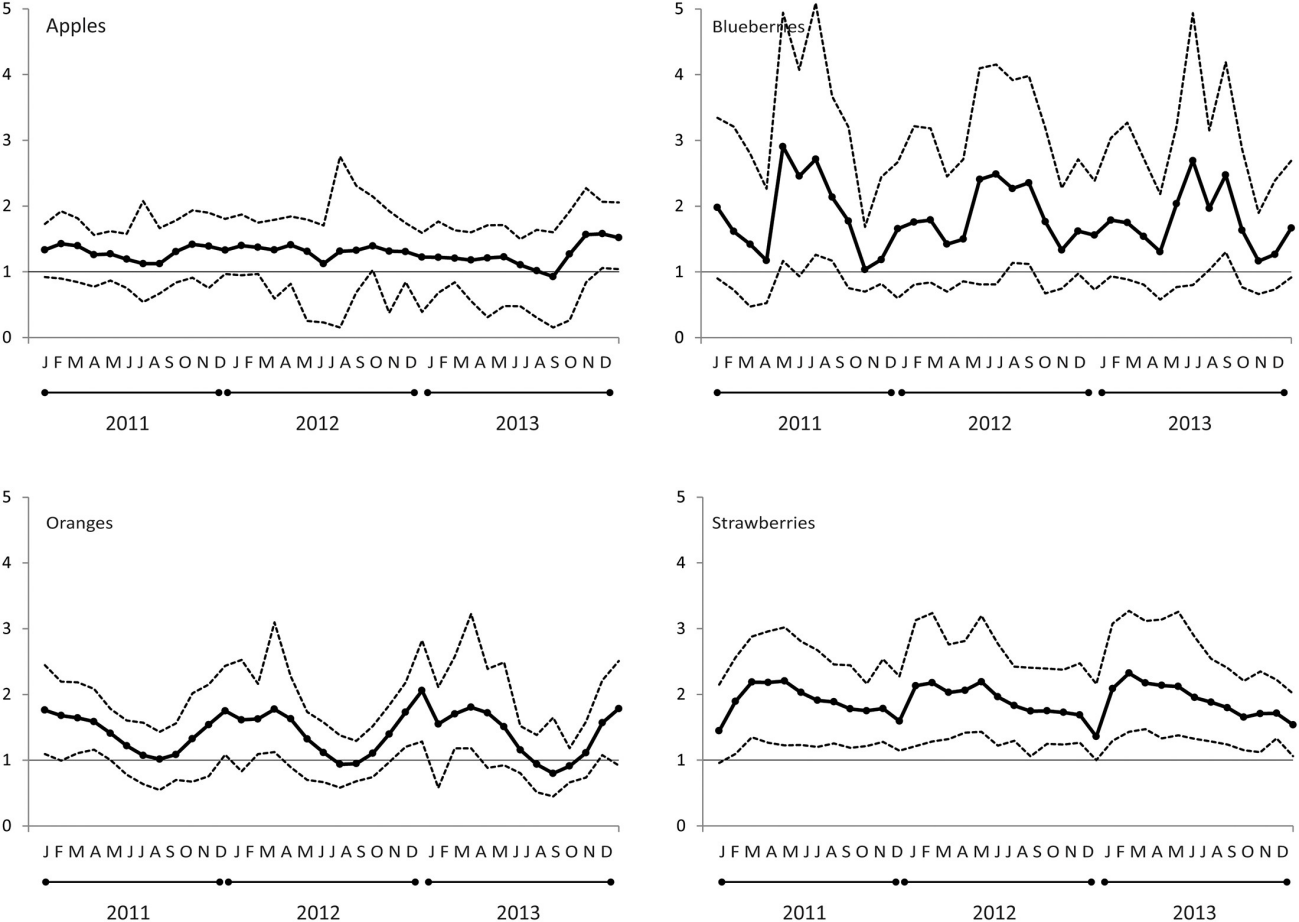
5th percentile, mean, and 95th percentile organic to conventional price ratios each month during the 2011–2013 period for apples, blueberries, oranges, and strawberries. Prices depicted in this graph are monthly national averages that are *not* weighted by sampled household projection factors. Hallam [61] found organic price premiums of 20 to 30% in OECD countries in the early 2000s. USDA-ERS [62] also analyzed prices for 18 fruits with 2005 data and found that the organic premium was less than 30% for most items. Blueberries were the anomaly; its price premium exceeded 100%.

In Fig 3 we give the national-level 5th percentile, mean, and 95th percentile organic to conventional fruit price ratios *by month* during the 2011 to 2013 period for apples, blueberries, oranges, and strawberries. The apple, orange, and strawberry price ratio trends are in line with Hallam’s [61] earlier finding that organic price premiums are 20 to 30% in OECD countries. USDA-ERS [62] also analyzed prices for 18 fruits with 2005 data and found that the organic premium was less than 30% for most items. In our data blueberry price ratios were the anomaly to these trends as its price premium exceeded 100% in the summer months. This figure also highlights the strong seasonal trend in fruit prices; a cycle that is hidden when we averaged fruit prices across months (S3 Table).

### Estimating household demand for organic fruit by household income class

To simulate the distributional impacts of an organic fruit subsidy we first had to estimate monthly household demand for organic fruit by household income class. We estimate these demand functions with three different methods. Each method complements each other as each method has unique statistical properties or assumes unique behavioral constraints. Therefore, the range in expected household monthly organic fruit consumption for each income class we derive is robust to various assumptions. Similar to Alcott et al. [47], we do not weight households by their projection factors when we estimate models of monthly household organic fruit consumption.

#### First estimation method: Separate equations

In our first estimation method we econometrically parameterize monthly conditional demand (positive) and unconditional demand (positive or zero), measured in ounces, for each fruit type × variety *i* and household income class *z* combination *separately* (*z* indexes the three household income classes we model, low, middle, and high). Therefore, in this first estimation method we do not assume that fruit consumption choices are made jointly nor do we impose any behavioral restrictions on household purchasing behavior.

The first estimation method’s unconditional expectation for ounces of fruit type × variety *i* bought by household-month *km* ∈ *z* is given by,

$$E\left[o_{ikm}\right]=P(e_{ikm}>0)E\left[o_{ikm}|e_{ikm}>0\right]+P(e_{ikm}=0)0$$ (1)

where *e*_*ikm*_ > 0 means household-month *km* ∈ *z* purchased a positive amount of *i*. The conditional demand term *E*[*o*_*ikm*_ | *e*_*ikm*_ > 0], representing expected ounces of *i* purchased by household-month *km* ∈ *z* given they buy a positive amount, is represented by the equation,

$$X_{km}\beta_{i}+c_{km}\sigma_{i}+P_{km}\theta_{i}+\gamma_{i}\lambda_{ikm}\left(X_{km}\delta_{i}+C_{km}\mu_{i}+P_{km}\omega_{i}\right)$$ (2)

where **P**_*km*_ is the set of 10 organic and conventional fruit prices household *k* faced in month *m*, *λ*_*ikm*_(**X**_*km*_*δ*_*i*_ + **C**_*km*_*μ*_*i*_ + **P**_*km*_*ω*_*i*_) is a Heckman-style correction term, and all other Greek letters represent model coefficients (the variables **X**_*km*_, **C**_*km*_ and **c**_*km*_ variables are explained at the beginning of the “Data” section). We include a Heckman-style correction in (2) because this subset of purchasing households may be a selected sample. The Heckman-style correction controls for the propensity of *km* ∈ *z* to purchase a positive amount of *i*.

Assuming the errors in the regression model designed to parameterize (1) are normally distributed, then Eq (1) is estimated with,

$$E\left[o_{ikm}\right]=P(e_{ikm}>0)E\left[o_{ikm}|e_{ikm}>0\right]+P(e_{ikm}=0)0$$ (3)

$$=\underset{P(e_{ikm}>0)}{\underset{︸}{\Phi \left(X_{km}\delta_{i}+C_{km}\mu_{i}+P_{km}\omega_{i}\right)}}\underset{E\left[o_{ikm}|e_{ikm}>0\right]}{\underset{︸}{\left[X_{km}\beta_{i}+c_{km}\sigma_{i}+P_{km}\theta_{i}+\gamma_{i}\underset{\lambda_{ikm}}{\underset{︸}{\frac{\phi \left(X_{km}\delta_{i}+C_{km}\mu_{i}+P_{km}\omega_{i}\right)}{\Phi \left(X_{km}\delta_{i}+C_{km}\mu_{i}+P_{km}\omega_{i}\right)}}}\right]}}$$ (4)

We estimate (3)-(4) in two stages. First, we use a probit over all household-month *km* ∈ *z* to parameterize *P*(*e*_*ikm*_ > 0). Then we use ordinary least squares (OLS) over household-month *km* ∈ *z* where *e*_*ikm*_ > 0 to estimate *E*[*o*_*ikm*_ | *e*_*ikm*_ > 0].

The predicted unconditional monthly ounces of *i* consumed by the representative *km*∈*z*, given by ${\hat{o}}_{iz}^{u1}\left(X_{km},c_{km},C_{km},P_{km}\right)$, is found by evaluating estimated,

$$\underset{\hat{P}(e_{ikm}>0)}{\underset{︸}{\Phi \left(X_{km}{\hat{\delta }}_{i}+C_{km}{\hat{\mu }}_{i}+P_{km}{\hat{\omega }}_{i}\right)}}\underset{\hat{E}\left[o_{ikm}|e_{ikm}>0\right]}{\underset{︸}{\left[X_{km}{\hat{\beta }}_{i}+c_{km}{\hat{\sigma }}_{i}+P_{km}{\hat{\theta }}_{i}+{\hat{\gamma }}_{i}\underset{{\hat{\lambda }}_{ikm}}{\underset{︸}{\frac{\phi \left(X_{km}{\hat{\delta }}_{i}+C_{km}{\hat{\mu }}_{i}+P_{km}{\hat{\omega }}_{i}\right)}{\Phi \left(X_{km}{\hat{\delta }}_{i}+C_{km}{\hat{\mu }}_{i}+P_{km}{\hat{\omega }}_{i}\right)}}}\right]}}$$ (5)

at mean **X**_*km*_, **c**_*km*_, **C**_*km*_, and **P**_*km*_ across all household-months *km* ∈ *z* (S4 Table). The ‘1’ in ${\hat{o}}_{iz}^{u1}$ indicates the first estimation method. The predicted conditional monthly ounces of *i* consumed by the representative *km*∈*z* where *e*_*ikm*_ > 0, given by ${\hat{o}}_{iz}^{c1}\left(X_{km},c_{km},C_{km},P_{km},{\hat{\lambda }}_{ikm}\right)$, is found by evaluating estimated $\hat{E}\left[o_{ikm}|e_{ikm}>0\right]$ at mean **X**_*km*_, **c**_*km*_, **C**_*km*_, and **P**_*km*_ for household-months *km* ∈ *z* where *e*_*ikm*_ > 0 (S5 Table). To see the estimated forms of ${\hat{o}}_{iz}^{c1}$ and ${\hat{o}}_{iz}^{u1}$, including estimated *δ*_*i*_, *μ*_*i*_, *ω*_*i*_, *β*_*i*_, *σ*_*i*_ and *ϕ*_*i*_, refer to the instructions for running the relevant Stata.do files in S5 Supporting information.

#### Second estimation method: Linquad

In the first econometric approach we assumed that expenditures on fruit type × variety *i* was exogenous to other fruit purchases and household budget. However, this many not be the case. Instead, consumers may allocate a portion of their income over a joint purchase of several fruit types and varieties. If this latter narrative better represents actual consumer behavior then we should estimate fruit purchases with a demand system where all fruit expenditures are determined jointly. In this case we adopted the LinQuad demand system [63–68]. This system models demand with a well-defined expenditure function and imposes several consumer theory restrictions, including homogeneity in prices and Slutsky substitution matrix symmetry. We selected LinQuad over other demand system estimation methods because LinQuad does not require the modeler to have household expenditure shares across all categories of consumables [69–70].

Under this method the unconditional and conditional expectations for ounces of *i* bought by household-month *km* ∈ *z* is given, respectively, by,

$$\begin{array}{r} E\left[o_{ikm}\right]=\Phi \left(X_{km}{\hat{\delta }}_{i}+C_{km}{\hat{\mu }}_{i}+P_{km}{\hat{\omega }}_{i}\right)f\left(X_{km}\beta_{i},c_{km}\sigma_{i},P_{km}\theta_{i},s_{km}\right)+\\ \gamma_{i}\phi (X_{km}{\hat{\delta }}_{i}+C_{km}{\hat{\mu }}_{i}+P_{km}{\hat{\omega }}_{i}) \end{array}$$ (6)

and

$$E\left[o_{ikm}|e_{ikm}>0\right]=f\left(X_{km}\beta_{i},c_{km}\sigma_{i},P_{km}\theta_{i},s_{km}\right)+\gamma_{i}{\hat{\lambda }}_{ikm}$$ (7)

where ${\hat{\lambda }}_{ikm}$, $\phi (X_{km}{\hat{\delta }}_{i}+C_{km}{\hat{\mu }}_{i}+P_{km}{\hat{\omega }}_{i})$, and $\Phi \left(X_{km}{\hat{\delta }}_{i}+C_{km}{\hat{\mu }}_{i}+P_{km}{\hat{\omega }}_{i}\right)$ are the same as the estimated functions found in (5). However, the function *f*(**X**_*km*_*β*_*i*_, **c**_*km*_σ_*i*_, **P**_*km*_θ_*i*_, *s*_*km*_) in Eqs (6) and (7) [71] is not found in the first estimation method. This function explains a households’ monthly consumption of ounces of *i* as a function of a household’s characteristics, fruit prices faces, and the household’s budget constraint,

$$\begin{array}{ll} o_{ikm} & =\alpha_{i}+X_{km}\beta_{i}+c_{km}\sigma_{i}+P_{km}\theta_{i}+\\ & \;\;\;\psi \left[s_{km}-P_{km}\alpha -X_{km}\beta P_{km}^{\prime }-c_{km}\sigma P_{km}^{\prime }-0.5P_{km}\theta P_{km}^{\prime }\right] \end{array}$$ (8)

where *s*_*km*_ refers to household *k*’s income in month *m* (*s*_*km*_ is a component of **X**_*km*_ but is highlighted with its own symbol due to its importance), and the bracket of Eq (8) is *km*’s budget constraint. The constraint in (8) limits expenditure on *i* by *km* ∈ *z* to be less than or equal to its monthly income *s*_*km*_. Following the LinQuad method, we use a seemingly unrelated regression (SUR) approach to estimate *f*(**X**_*km*_*β*_*i*_, **c**_*km*_σ_*i*_, **P**_*km*_θ_*i*_)’s parameters *β*, *σ*, *α*, *θ*, and *ψ* across all *i* jointly (less organic-other and conventional-other) using *all* household-months *km* ∈ *z* with imposed symmetry in the price coefficients and homogeneity in prices and income (we allow for correlation in the regression errors across equations). Notice the other difference between the LinQuad method and the separate equation method described above: with the LinQuad method *E*[*o*_*ikm*_ | *e*_*ikm*_ > 0] is estimated with all *km* ∈ *z*, not just those where *e*_*ikm*_ > 0.

The predicted unconditional monthly ounces of *i* consumed by a representative *km*∈*z*, given by ${\hat{o}}_{iz}^{u2}\left(X_{km},c_{km},C_{km},P_{km},s_{km}\right)$, is found by evaluating the estimated (6) at mean **X**_*km*_, **c**_*km*_, **C**_*km*_, and **P**_*km*_ across all household-months *km* ∈ *z* (S4 Table). The ‘2’ in ${\hat{o}}_{iz}^{u2}$ indicates the second estimation method. The predicted conditional monthly ounces of *i* consumed by a representative *km*∈*z*, given by ${\hat{o}}_{iz}^{c2}\left(X_{km},c_{km},P_{km}\right)$, is found by evaluating the estimated (8) at mean **X**_*km*_, **c**_*km*_, **C**_*km*_, and **P**_*km*_ for *km* ∈ *z* where *e*_*ikm*_ > 0 (S5 Table). To see the estimated forms of ${\hat{o}}_{iz}^{u2}$ and ${\hat{o}}_{iz}^{c2}$, including estimated *β*, *σ*, *α*, *θ*, and *ψ*, refer to the instructions for running the relevant Stata.do files in S5 Supporting information.

#### Third estimation method: LASSO

For the third demand estimation method we use the ML technique known as LASSO (least absolute shrinkage and selection operator). Unlike the econometric techniques, LASSO is not based on a data generating theory. Instead the “data” determines the final set of predictors (the universe of *possible* explanatory variables is the only place for economic intuition in these methods).

Economists have rarely used ML techniques like LASSO because they generate ‘agnostic’ predictive models rather than models of inference that allow for the testing of economic theory. For example, economists assume prices explain demand for a product and therefore include prices in the product’s demand models. In contrast, the LASSO technique may find that some (or even all) prices are not particularly predictive of observed demand and therefore drop these prices from the final predictive heuristic in order to minimize mean squared prediction error (the objective of LASSO estimation). However, recall the point of this research is to *predict* as accurately as possible how an organic food subsidy would play out across household income brackets in the US. Therefore, using a predictive algorithm that might ignore basic economic intuition is consistent with our research motives (see [72–73] for other examples of counterfactual analysis with ML techniques). Further, if the two econometric estimation methods described above mis-specify the data generating process or behavioral constraints across consumers of organic fruit then the LASSO demand estimates could be the most accurate depiction of household demand for organic fruit.

Like the first estimation method, we use LASSO to *separately* predict unconditional and conditional demand for each fruit type × variety *i* in income group *z*. Also like the first estimation method, we estimate the LASSO-conditional demand function only using observations where *e*_*ikm*_ > 0 (the second estimation method is a system where both conditional and unconditional demand is estimated over *all* observations). However, unlike the first two estimation methods, we did not predict representative household monthly demand for *i* at the mean regressor values with our LASSO model. Instead, with the LASSO method we predicted representative unconditional (conditional) household-month demand for *i* by predicting quantity demanded for each *km*∈*z* (each *km*∈*z* where *e*_*ikm*_ > 0) and then taking the mean of the vector of predicted quantities (the mean of the predictions rather than the prediction over means).

In the first step of LASSO estimation we find the model coefficients that best explain whether or not a household-month *km*∈*z* purchased *i*. This is done by maximizing the log likelihood function of the linear logistic model,

$${\text{max}}_{\pi_{i}}\frac{1}{N_{z}}\left[\sum_{km=1}^{N_{z}}\left\{I\left(e_{ikm}=0\right)\text{log}\;\rho \left(W_{km}\right)+I\left(e_{ikm}>0\right)\text{log}\left(1-\rho \left(W_{km}\right)\right)\right\}-\mu_{i}\sum_{j=1}^{v}\left|\pi_{ij}\right|\right]$$ (9)

where *N*_*z*_ is the set of all *km*∈*z*, *I*(*e*_*ikm*_ = 0) indicates a *km*∈*z* observation where *i* was not purchased, *I*(*e*_*ikm*_ > 0) indicates a *km*∈*z* observation where *i* was purchased, $W_{km}\;=\;[X_{km}^{'}C_{km}^{'}P_{km}]$ is a vector of *v* standardized candidate predictors for the binary consumption of *i*, $\rho (W_{km})\;=\;\frac{1}{1+exp\;(-W_{km}\pi_{i})}$ is the probability that *i* is not purchased by *km*∈*z*, the shrinkage penalty $\sum_{j\;=\;1}^{v}\left|\pi_{ij}\right|$ is the sum of independent variable coefficients for purchases of *i* by *km*∈*z*, and *μ*_*i*_ is the tuning parameter that adjusts the severity of the shrinkage penalty. The shrinkage penalty in Eq (9) is a kinked function of **π**_*i*_. Therefore, the LASSO tends to set some model coefficients to zero.

The primes on **X**_*km*_ and **C**_*km*_ in the Eq (9)’s vector **W**_*km*_ indicate that these household-month variable vectors are different than the **X**_*km*_ and **C**_*km*_ variable vectors used with estimation methods 1 and 2 in two ways. First, the **X**_km_ and **C**_*km*_ vectors used in estimation methods 1 and 2 are comprised of a set of author-selected household-month and market variables found in the Consumer Panel dataset. In contrast, $X_{km}^{'}$ and $C_{km}^{'}$ include *all* of the household-month variables available from the Consumer Panel dataset. Second, the household-month variables in **X**_*km*_ and **C**_*km*_ are simplified representations of the more complex raw data found in the Consumer Panel dataset. In $X_{km}^{'}$ and $C_{km}^{'}$ we use the more complex raw data. For example, in the Consumer Panel dataset a household-month is placed into one of nine categories regarding the number and mix of children in the household. In **X**_*km*_ this variable was reduced to a binary variable that indicated whether household *k* had one or more children in month *m* or not. In $X_{km}^{'}$ all nine children categories are potential predictors. Further, in the **C**_km_ vector used in estimation methods 1 and 2 the rural-urban continuum code (RUCC) categories are reduced to a dummy representation [59]. In $C_{km}^{'}$ all seven RUCC categories are used.

The second step in predicting monthly household demand for fruit type × variety *i* in income group *z* with the LASSO model involves minimizing the log-likelihood function,

$${\text{min}}_{\vartheta_{i}}\left[\frac{1}{T_{iz}}\sum_{km=1}^{T_{iz}}{\left(o_{ikm}-w_{ikm}\vartheta_{i}\right)}^{2}+\mu_{i}\sum_{j=1}^{q}\left|\vartheta_{ij}\right|\right]$$ (10)

where *T*_*iz*_ is the set of set of *km*∈*z* where *e*_*ikm*_ > 0, $w_{ikm}\;=\;[X_{km}^{'}C_{km}^{'}P_{km},\;\rho_{ikm}]$ is a matrix of *q* standardized candidate predictors for the consumption of *i*, vector **ϑ**_*i*_ contains linear model coefficients for *i*, and *μ*_*i*_ is the tuning parameter, as described above.

Note that **w**_*ikm*_ includes *km*∈*z*’s probability of purchasing *i* in a given month. Because model (10) is agnostic regarding the distributional shape of a model’s errors, we cannot use the inverse mills ratio to correct for self-selection bias when estimating and predicting demand for fruit *i* as we did with the econometric methods (the inverse mills ratio assumes normally distributed errors). Instead we use *ρ*_*ikm*_ itself as a propensity score in the LASSO conditional demand function to control for self-selection (the coefficient on this variable is separately identified in the linear second stage model because the first stage is estimated using the non-linear logistic function).

To estimate (9)-(10) for *i* we first solved (9) over all *km*∈*z*. Then we calculated ${\hat{\rho }}_{ikm}\;=\;\frac{1}{1+exp\;(-W_{km}{\hat{\pi }}_{i})}$ for each *km*∈*z*. Next, we solved Eq (10) for *i*, i.e., generated *${\hat{\vartheta }}_{iz}$,* using the data $w_{ikm}\;=\;[X_{km}^{'}C_{km}^{'}P_{km},\;{\hat{\rho }}_{ikm}]$ from the set of *km*∈*z* where *e*_*ikm*_ > 0. The LASSO-predicted unconditional monthly household demand for *i* was found by generating ${\hat{o}}_{ikm}^{u3}\;=\;{\hat{\rho }}_{ikm}(w_{ikm}{\hat{\vartheta }}_{iz})$ across each *km*∈*z* and then taking the average of ${\hat{o}}_{ikm}^{u3}$. The LASSO predicted conditional demand was found by generating ${\hat{o}}_{ikm}^{c3}\;=\;w_{ikm}{\hat{\vartheta }}_{iz}$ across all the set of *km*∈*z* where *e*_*ikm*_ > 0 and then taking the average of ${\hat{o}}_{ikm}^{c3}$.

The standard errors for estimates of *π*_*ij*_ and *θ*_*ij*_ are bootstrapped by replicating the joint solution to Eqs (9) and (10) 100 times. In each replicate, the sample is randomly drawn with replacement, so while the sample size is the same each time, the households represented in the dataset are different in each replication. See S6 Table for the means over a select number of variables found in **w**_*ikm*_. To see the estimated forms of ${\hat{o}}_{iz}^{u3}$ and ${\hat{o}}_{iz}^{c3}$, including estimated ${\hat{\rho }}_{ikm}$ and ${\hat{\vartheta }}_{iz}$, refer to the instructions for running the relevant R scripts in S5 Supporting information.

#### Simulating the total impact of organic food subsidization on representative households

First, as described above, we predicted monthly unconditional and conditional household quantity demanded for a representative household in each income class. Second, we reduced the organic fruit prices a representative household in income class *z* faced by 10% (given by $\;{\tilde{P}}_{z}$). In the econometric-based methods this meant reducing the *z*’s mean prices by 10%. With the LASSO this meant reducing all *z* households’ observed prices by 10%. Third, we predicted the amount of money the household would save with the subsidy if it continued to purchase organic fruit at predicted pre-subsidy levels (infra-marginal gain due to the subsidy). Let the unconditional and conditional infra-marginal gains due to the subsidy at representative household *z* using estimation method *j* be given by ${\hat{o}}_{iz}^{uj}(.|P_{z})(P_{iz}-{\tilde{P}}_{z})$ and ${\hat{o}}_{iz}^{cj}(.|P_{z})(P_{iz}-{\tilde{P}}_{z})$, respectively. Fourth, we predicted the representative household’s change in monthly unconditional and conditional quantity demanded. Let the change in unconditional and conditional quantity demanded of *i* at representative household *z* using estimation method *j* be given by $\Delta {\hat{o}}_{iz}^{uj}={\hat{o}}_{iz}^{uj}(.|{\tilde{P}}_{z})-{\hat{o}}_{iz}^{uj}(.|P_{z})$ and $\Delta {\hat{o}}_{iz}^{cj}\;=\;{\hat{o}}_{iz}^{cj}(.|{\tilde{P}}_{z})-{\hat{o}}_{iz}^{cj}(.|P_{z})$, respectively. The representative household’s change in purchasing behavior and infra-marginal gain together indicate the total impact of the subsidy on the household.

Finally, we estimated each *z*’s unconditional and conditional purchase and expenditure elasticities across each *i* and each estimation method *j*. A purchase elasticity for organic fruit *i* measures the percentage change in ounces of *i* bought per month for each 1% increase in the prices of *all* organic fruit. An expenditure elasticity for organic fruit *i* measures the percentage change in dollars spent per month on *i* for each 1% increase in the prices of *all* organic fruit. Therefore, in this case, a negative (positive) elasticity means predicted purchases or expenditures *increased* (decreased) with the organic fruit price subsidy. See Tables 4 and 5 for all predicted values of ${\hat{o}}_{iz}^{uj}(.|P_{z})(P_{iz}-{\tilde{P}}_{z}),\;{\hat{o}}_{iz}^{cj}(.|P_{z})(P_{iz}-{\tilde{P}}_{z}),\;\Delta {\hat{o}}_{iz}^{uj},\;\Delta {\hat{o}}_{iz}^{cj}$, and unconditional and conditional purchase and expenditure elasticities.

**Table 4:** Predicted conditional reaction to an organic fruit price subsidy across household types. Under the subsidy plan all organic fruit prices are 90% of observed 2011–2013 prices. Reactions are calculated for a representative lower, middle, and upper income class household. All dollar values are measured in Dec. 2013 $.

	Monthly savings ($) on original purchase w/ subsidy			Change in oz. purchased per month			Monthly purchase elasticity			Monthly expenditure elasticity		
Household Income Class	Low	Middle	High	Low	Middle	High	Low	Middle	High	Low	Middle	High
**Est. method**	**Apples**											
Separate equations	0.70 (0.05)	0.71 (0.07)	0.75 (0.03)	12.68 (4.57)	21.49 (5.52)	10.46 (2.51)	-1.75 (0.54)	-2.94 (0.78)	-1.36 (0.28)	-0.58 (0.49)	-1.65 (0.70)	-0.22 (0.25)
LinQuad	0.82 (0.22)	1.19 (0.20)	0.86 (0.05)	18.42 (12.80)	26.92 (14.6)	6.44 (4.37)	-2.12 (1.64)	-2.14 (0.85)	-0.71 (0.46)	-0.91 (1.48)	-0.93 (0.76)	0.36 (0.42)
LASSO	0.80 (0.05)	0.66 (0.02)	0.71 (0.01)	0.00 (4.49)	25.00 (4.61)	10.26 (2.84)	0.00 (0.52)	-2.94 (0.48)	-1.25 (0.32)	1.00 (0.59)	-2.49 (0.67)	-0.34 (0.37)
	**Blueberries**											
Separate equations	0.85 (0.06)	0.77 (0.02)	0.81 (0.01)	-0.07 (0.96)	0.27 (0.40)	0.38 (0.23)	0.04 (0.53)	-0.16 (0.23)	-0.22 (0.13)	1.03 (0.47)	0.86 (0.21)	0.80 (0.12)
LinQuad	1.09 (0.17)	0.97 (0.05)	1.04 (0.43)	0.45 (0.62)	0.15 (0.18)	0.80 (0.16)	-0.20 (0.28)	-0.08 (0.10)	-0.38 (0.08)	0.82 (0.25)	0.93 (0.09)	0.65 (0.07)
LASSO	0.71 (0.08)	0.62 (0.02)	0.64 (0.01)	1.50 (1.45)	1.06 (0.41)	0.83 (0.32)	-0.82 (1.00)	-0.60 (0.23)	-0.48 (0.18)	-0.19 (1.88)	0.12 (0.26)	0.24 (0.21)
	**Oranges**											
Separate equations	0.59 (0.05)	0.58 (0.02)	0.60 (0.03)	-1.93 (4.05)	-0.97 (1.21)	-0.01 (1.77)	0.26 (0.55)	0.14 (0.17)	0.00 (0.24)	1.24 (0.50)	1.12 (0.15)	1.00 (0.22)
LinQuad	0.60 (0.15)	0.62 (0.06)	0.63 (0.06)	3.37 (8.69)	-2.62 (2.83)	-3.60 (2.43)	-0.47 (1.23)	0.36 (0.37)	0.49 (0.32)	0.58 (1.10)	1.32 (0.33)	1.44 (0.29)
LASSO	0.60 (0.05)	0.57 (0.02)	0.55 (0.02)	-0.47 (4.16)	-0.43 (1.53)	0.00 (1.75)	0.06 (0.54)	0.06 (0.22)	0.00 (0.25)	1.06 (0.49)	1.05 (0.20)	1.00 (0.23)
	**Strawberries**											
Separate equations	0.60 (0.03)	0.60 (0.01)	0.65 (0.01)	0.81 (0.79)	0.26 (0.27)	0.83 (0.27)	-0.32 (0.31)	-0.10 (0.10)	-0.31 (0.10)	0.71 (0.28)	0.91 (0.09)	0.72 (0.09)
LinQuad	0.76 (0.14)	0.75 (0.04)	0.78 (0.03)	0.54 (1.20)	-1.89 (0.43)	-0.03 (0.27)	-0.19 (0.42)	0.67 (0.14)	0.01 (0.09)	0.83 (0.38)	1.61 (0.13)	1.01 (0.08)
LASSO	0.59 (0.03)	0.61 (0.01)	0.63 (0.01)	0.00 (0.96)	0.46 (0.28)	0.48 (0.26)	0.00 (0.37)	-0.17 (0.11)	-0.18 (0.10)	1.00 (0.35)	0.84 (0.10)	0.83 (0.09)

**Table 5:** Predicted unconditional reaction to an organic fruit price subsidy across US three household types. Under the subsidy plan all organic fruit prices are 90% of observed 2011–2013 prices. Reactions are calculated for a representative lower, middle, and upper income class household. All dollar values are measured in Dec. 2013 $.

	Monthly savings ($) on original purchase w/ subsidy			Change in oz. purchased per month			Monthly purchase elasticity			Monthly expenditure elasticity		
Household Income Class	Low	Middle	High	Low	Middle	High	Low	Middle	High	Low	Middle	High
**Est. method**	**Apples**											
Separate equations	0.001 (0.0006)	0.001 (0.0011)	0.002 (0.0004)	0.0036 (0.0098)	0.0053 (0.0135)	-0.0052 (0.0069)	-0.36 (0.89)	-0.40 (0.91)	0.27 (0.37)	0.67 (0.81)	0.64 (0.82)	1.24 (0.33)
LinQuad	0.001 (0.0003)	0.001 (0.0002)	0.002 (0.0001)	0.110 (0.0140)	0.015 (0.0120)	-0.010 (0.0070)	-1.02 (1.49)	-0.97 (0.67)	0.45 (0.36)	0.08 (1.34)	0.13 (0.61)	1.40 (0.32)
LASSO	0.002 (0.0002)	0.003 (0.0001)	0.004 (0.0001)	0.000 (0.19)	0.230 (0.05)	0.130 (0.04)	0.00 (0.52)	-2.68 (0.39)	-1.26 (0.33)	1.00 (0.55)	-2.11 (0.52)	-0.28 (0.35)
	**Blueberries**											
Separate equations	0.001 (0.0005)	0.001 (0.0002)	0.003 (0.0004)	0.0006 (0.0017)	0.0017 (0.001)	-0.0006 (0.0014)	-0.29 (0.87)	-0.71 (0.39)	0.10 (0.27)	0.74 (0.78)	0.36 (0.35)	1.09 (0.24)
LinQuad	0.001 (0.0002)	0.001 (0.0001)	0.003 (0.0002)	0.010 (0.0010)	0.010 (0.0006)	0.004 (0.0009)	-0.42 (0.56)	-2.59 (0.26)	-0.60 (0.14)	0.62 (0.51)	-1.33 (0.23)	0.46 (0.13)
LASSO	0.002 (0.0002)	0.002 (0.0001)	0.005 (0.0002)	0.03 (0.05)	0.007 (0.003)	0.010 (0.005)	-0.93 (0.83)	-0.55 (0.22)	-0.45 (0.18)	-0.27 (1.01)	0.22 (0.24)	0.32 (0.20)
	**Oranges**											
Separate equations	0.001 (0.0007)	0.001 (0.0002)	0.002 (0.0004)	0.0053 (0.0176)	0.001 (0.0042)	0.0154 (0.0107)	-0.39 (1.26)	-0.11 (0.48)	-0.82 (0.52)	0.65 (1.14)	0.90 (0.43)	0.27 (0.47)
LinQuad	0.001 (0.0004)	0.001 (0.0001)	0.002 (0.0002)	0.014 (0.0210)	0.0002 (0.0050)	-0.001 (0.0070)	-1.09 (1.83)	-0.02 (0.56)	0.03 (0.42)	0.02 (1.65)	0.98 (0.5)	1.03 (0.38)
LASSO	0.001 (0.0001)	0.001 (0.00004)	0.001 (0.00005)	-0.030 (0.59)	-0.0008 (0.009)	0.000 (0.010)	0.17 (0.51)	0.03 (0.22)	0.00 (0.28)	1.15 (0.47)	1.02 (0.20)	1.00 (0.23)
	**Strawberries**											
Separate equations	0.002 (0.0007)	0.003 (0.0004)	0.007 (0.0009)	0.015 (0.0078)	0.031 (0.0052)	0.048 (0.0088)	-1.84 (0.77)	-2.81 (0.31)	-1.83 (0.26)	-0.65 (0.69)	-1.53 (0.28)	-0.64 (0.23)
LinQuad	0.002 (0.0010)	0.002 (0.0002)	0.007 (0.0004)	0.020 (0.0100)	0.045 (0.0050)	0.050 (0.0100)	-3.59 (2.32)	-5.11 (0.73)	-2.10 (0.43)	-2.23 (2.10)	-3.60 (0.66)	-0.89 (0.39)
LASSO	0.003 (0.0002)	0.004 (0.0001)	0.008 (0.0002)	0.000 (0.04)	0.010 (0.007)	0.010 (0.008)	0.00 (0.35)	-0.21 (0.11)	-0.16 (0.09)	1.00 (0.33)	0.81 (0.10)	0.85 (0.09)

Please note that we do not specify the subsidy that would lead to a 10% reduction in the retail prices of all organic fruit. The source of the subsidy is outside the scope of our study. For example, prices that consumers face can be reduced by a point of purchase subsidy or a production subsidy that generates lower production costs. In the end, whatever form it would take, we assume that this subsidy reduces the prices of all organic fruit by 10% from 2011–2013 levels.

## Results and discussion

### Impact of independent variables on the propensity to consume and amount of consumption

Below we do not discuss the impact of independent variables on the propensity of a household to consume organic fruit or the estimated impacts of small changes in independent variable values on organic fruit consumption. Instead the interested reader can generate all marginal effects by running the computer code and datasets provided in the SI. However, prior to discussing the subsidy’s expected impact on households’ inframarginal savings and consumption and expenditure behavior, we briefly note the impact independent variables had on the propensity of a household to consume organic fruit and the amount of organic fruit consumed in the estimated LASSO model. The number of times LASSO bootstrap replicates generate non-zero LASSO coefficients offers some information on the importance of the various independent variables in explaining organic fruit consumption behavior (S7 and S8 Tables).

Fruit prices, household income, number of children in the household, household race indicators, and the rural-urban continuum classification categories consistently generated non-zero LASSO coefficients across replicates in most organic fruit *selection* models. At the *quantity* stage of consumption, LASSO replicates consistently generated non-zero price variable, income variable, and race variable coefficients over middle and rich household demand models. In contrast, prices and household-level variables did not consistently predict fruit quantity consumed in poor households. Overall, the LASSO replicates indicate that the variables typically used to predict or explain household consumption of organic food are least relevant among the poorest US households. This suggests that either 1) the poor had difficulty accessing organic fruit from 2011–2013 and therefore prices and household characteristics are irrelevant to their choices, 2) the decision to purchase organic fruit among poorer households is a function of omitted variables, or 3) some combination of both of these issues.

### Impact of subsidy on conditional purchases and expenditures

For households that already purchase some amount of organic fruit *i* in a given month (i.e., conditional demand), an organic fruit subsidy would compel them to, on average, consume more organic apples, blueberries, and strawberries each month than they had previously, all else equal (see the monthly purchase columns in Table 4). Between these three fruits, conditional organic apple consumption would increase the most (in a relative sense) (Table 4, S4 Fig). Conversely, the conditional consumption of organic oranges would, on average, fall or stay flat, all else equal. Of the four fruits we focus on, organic apples are the only fruit type that habitual buyers of organics, regardless of income class, would, on average, spend more on after the subsidy than before (i.e., only organic apple conditional expenditure elasticities tend to be negative; Table 4, S5 Fig).

The conditional inframarginal gains from the subsidy are similar across household and fruit types (Table 4). Regardless of estimation technique or fruit type, the lower income class household often saves just as much or more on the inframargin due to the subsidy as the middle or upper-class household. These results indicate that among households that already purchase some amount of organic fruit *i* in a given month, pre-subsidy purchase patterns of *i* were not correlated with household income.

Further, there is no clear pattern in the subsidy’s impact on conditional purchases across income classes (see the purchase elasticities in Table 4 and S4 Fig). Across all three estimation techniques, the middle-income class household would, in a relative sense, increase their conditional consumption of organic apples as much or more than the other two household types, all else equal. Further, relative conditional purchases of blueberries would generally (but not universally) increase the most, on average, at the high-income class household. Conversely, we found that the representative lower income household would, on average, be as responsive if not more responsive to the subsidy than the representative high-income class household in terms of conditional organic orange and strawberry purchases (Table 4, S4 and S5 Figs). In conclusion, relative reaction to the subsidy is not consistently different across income types in households that already buy organic fruit (conditional demand).

Therefore, given similar pre-subsidy consumption patterns and no clear differences in purchase elasticities across household types that already buy organic fruit, the organic fruit subsidy would not be re-distributive at the consumption stage among the cohort of American households that already buy organic fruit. Of course, this assumes that no household class is particularly responsible for funding of the subsidy. If, for example, the subsidy was largely funded by the richer habitual buyers of organic fruit then the subsidy would re-distribute some welfare from richer to poorer households.

### Impact of the subsidy on unconditional purchases and expenditures

Considering all households, not just those that already buy organic fruit *i* in a given month (i.e., unconditional demand), an organic fruit subsidy would compel them, on average, to consume more organic blueberries and strawberries each month than they had previously, all else equal (Table 5, S4 Fig). In fact, several predictions of the relative change in unconditional quantity demanded of organic strawberries are the largest relative changes we observe across all subsidy counterfactuals. Further, across all unconditional organic fruit-estimation method reactions we model, only the econometrically-derived predictions of change in organic strawberry quantity demanded indicates that typical US households, regardless of income class, would unconditionally spend more on organic fruit after the subsidy than before (see the unconditional monthly expenditure elasticity graphs in S4 Fig). Almost all other unconditional expenditure elasticity predictions indicate that a 10% drop in organic fruit prices would mean that a representative US household would (unconditionally) spend *less* on of organic fruit *i* than before despite buying more of *i* in reaction to the subsidy. This pattern holds across all three income types (recall these results only hold for organic fruit purchased via a UPC).

Except for organic apples, unconditional purchase elasticities are larger, in absolute terms, than their conditional analogs. Further, again except for organic apples, unconditional expenditure elasticities are closer to zero (or in some cases, more negative) than their conditional analogs. This indicates that the subsidy would have a relatively larger impact on the purchasing patterns of typical US households than the subset of US households that already buy organic fruit (conditional demand). In other words, our analysis suggests that the subsidy would change the organic fruit buying behavior of the casual consumer more, in a relative sense, than it would the habitual consumer of organic fruit.

We found that a 10% subsidy would affect unconditional inframarginal savings across income classes differently. Specifically, upper income households typically would save more on their pre-subsidy organic fruit purchases given the subsidy than their lower and middle-income peers. This outcome simply reflects that when considering all households, not just habitual buyers of organic fruit, a typical rich household already buys more organic fruit than the typical household from the other two classes (Table 2). While *one* rich household’s infra-marginal gains due to the subsidy are very small relative to its representative peers—less than one cent per month across all fruits—if we sum this difference across all US households the aggregate differential across income class is significant. For example, using the data on the number of US households in each income class from Table 3 and the inframarginal gain predictions for strawberries from Table 5 we found that rich American households would gain approximately $2.65 million more in inframarginal savings per year than lower income American households. This is just considering strawberries.

Unlike conditional response to the subsidy, unconditional demand for organic fruit is such that a 10% organic fruit price subsidy would, on average, generate relatively different reactions across income class. Our analysis suggests that the subsidy would generally cause monthly organic fruit consumption at the representative poor or middle-income class household to increase relatively more than at the rich household, all else equal (Table 5, S4 and S5 Figs). This is unequivocally the case for organic apples, blueberries, and strawberries where, across all estimation methods, the lower or middle income representative households always has a larger (more negative) expenditure elasticity for these three fruits than the rich representative household.

In conclusion, considering all households, not just those that already buy organic fruit, their overall reaction to the subsidy is differentiated by income class. First, given that they already tend to buy more organic fruit than other household types, the bulk of unconditional inframarginal savings would accrue to upper income class households. However, rich households would not react as strongly to the subsidy as the other two household types; across almost all fruit and demand estimation technique combinations we found the largest (more negative) unconditional monthly purchase elasticity at the representative poor and/or middle-income class household. Whether the subsidy re-distributes unconditional surplus would ultimately depend on the tax incidence of the funds used to support the subsidy. A broadly applied tax or one that mostly relied on contributions from wealthier households would mean a re-distribution of surplus to poorer and middle-class households.

### Comparing estimation methods

A comparison of subsidy impacts across the three estimation techniques reveals three discernable patterns. First, the conditional inframarginal savings generated with the LinQuad system is always a bit higher than the conditional inframarginal savings generated with the Heckman-like and LASSO single equations, typically by about 10% to 25%. In other words, conditional organic fruit demand at pre-subsidy prices is always a bit higher according to the system-estimated demand equations than we predict with the other two methods. Second, the LASSO equations almost always find larger unconditional inframarginal savings than the other two methods (the exception to this trend is unconditional organic orange demand), but the difference is typically less than a penny for each fruit category, on average. Third, the LASSO-generated elasticity predictions almost always have standard errors that are as small or are smaller than the standard errors on elasticity predictions generated by the other two methods. However, we do not find that purchase and expenditure elasticities generated with one method are systematically smaller or larger than the purchase and expenditure elasticities generated with the other two methods.

As we mentioned above, empirical economists have rarely used ML techniques like LASSO because they generate ‘agnostic’ predictive models rather than models of inference. However, because our aim was to predict household reaction to a subsidy we were less interested in inference and therefore open to models primarily used for prediction. We were concerned that the LASSO-generated demand equations would include many fruit price variable coefficients equal to 0. If this had happened we would either have to conclude that fruit prices largely do not play a role in organic fruit consumption (highly doubtful) or that the LASSO method was not appropriate given our research aims. However, for the most part, however, LASSO-estimated demand equation did include non-zero price coefficients, particularly in the apple, blueberry, and strawberry selection stages (Eq (9)). Own organic and conventional prices as opposed to other fruit prices were particularly likely to have non-zero price coefficients (S7 and S8 Tables). In the few LASSO-estimated demand equations where prices were not found to be very predictive of purchasing behavior—this was the case for several low-income household demand equations and for several organic orange demand equations regardless of income class—we, not surprisingly, found LASSO-estimated purchase elasticities of zero or near zero and expenditure elasticities of one or near one.

## Conclusions

We predicted the impact of an organic fruit subsidy on patterns of organic fruit consumption and expenditures across US households of different income classes. We were particularly interested to determine if the subsidy would, on average, favor households from a certain income class. If we estimated significantly larger inframarginal savings and a particularly elastic response to the subsidy in one class it would suggest that the subsidization would re-distribute some social welfare to that class, especially if the revenue for the subsidy came from the general taxpayer.

In contrast to other CT subsidization, which has been found to be strongly regressive at the consumption stage, such as roof-top solar in Australia [42] or electric cars in the US [45,74], we find evidence that organic fruit subsidization in the US would, if anything, change the relative consumptive behavior of poor and middle-class households more than that of richer households. Of course, because richer households in the US tend to buy more organic fruit than poorer households already, the inframarginal savings from the subsidy would tend to be larger in richer households. However, this inframarginal savings advantage enjoyed by richer households in general disappears among the smaller subset of households that habitually buy organic fruit (conditional demand): conditional inframarginal savings generated by fruit subsidies are relatively equal across all three income classes and fruit types.

While we find that most households would buy a bit more organic fruit with a price subsidy, whether the subsidy would generate net welfare across households is an open question. Ideally, we would evaluate a representative household’s welfare gains from the subsidy using an explicit household utility function. However, the Heckman-like and LASSO single equation estimation methods do not specify a utility function and estimating welfare changes with the LinQuad system proved problematic due to the large number of zero demanders. Of course, consumer surplus is not the only benefit the subsidy would create. The subsidy could also generate some positive farm worker and consumer health, environmental, and rural equity externalities via the promotion of a CT. For example, if organic food is healthier and more nutritious to eat than conventional fruit or other foods, then a subsidy would also promote better health among the wider US population. On the negative side, raising revenue for the subsidy would generate dead weight loss in the US economy that would need to be set against the potential welfare gain of any positive externalities. It is our hope that future research addresses questions over the net welfare impact of organic food subsidization.

Our approach to estimating the impact of organic fruit subsidization across different household income classes is limited in several ways. First, data on price paid, weight or number of fruit items bought, or the organic / conventional status of the fruit for purchases not completed with a UPC can be missing or unreliable. Therefore, we did not include non-UPC fruit purchases in our dataset. Not only does this mean that our dataset misses all fruit purchases made at grocery stores that did not involve the use of a UPC, but fruit purchases made at delis, farmers’ markets, road-side stalls, restaurants, etc., are also largely missing from our dataset. (This latter issue is less concerning given, as we mentioned above, 92 percent of organic food is purchased through grocery stores and natural food stores [75].) It is not clear how different our simulated policy impacts would be if we included fruit purchased without a UPC in our fruit dataset. For example, while approximately 40% of *all* fresh produce is bought with a UPC [47], if 20% of organic fruit is bought with a UPC but 50% of conventional fruit is bought with a UPC then our simulated results could be biased.

Second, we estimated average monthly fruit purchases each household faced by using observed prices from the household’s home market. When no prices were observed relevant prices were imputed (S2 Supporting information). This process introduces additional unmeasured error into our estimates. However, given that the LASSO model usually assigned non-zero coefficients to the price variables and most price elasticities had expected signs it appears that this additional error did not eliminate the role that prices should and did have in explaining consumer behavior over organic fruit. We must note, however, that LASSO estimates over data with measurement error are prone to attenuation bias (estimates are closer to 0 than they really are [76]).

Third, when estimating organic fruit consumption we do not weight households by their projection factors. Therefore, it is not clear how well the estimated demand functions and policy simulations capture the universe of US households rather than just the set of sampled households. If we assume the estimated demand functions are representative of the universe of US households we can measure the error in not using the projection factors by comparing simulated estimated representative household demand for fruit *I* with and without projection factors. When we average predicted monthly consumption of *i* across all *km*∈*z* with and without projection factor weights the predictions are generally within five to ten percent of one another (S9 and S10 Tables). The only exceptions to this pattern are found in some of the unconditional demand estimates across poor households. This finding adds weight to our earlier conclusion that our models do less well in describing demand for organic fruit among the poorer US households. Otherwise, assuming the estimated demand functions are representative of the universe of US households, our policy simulations are a reasonable guide to the impact of fruit subsidization across the US, not just across the sampled households.

Finally, we assume that monthly price fluctuations in fruit over the 2011–2013 are determined by exogenous supply fluctuations. To the extent that prices are endogenous, our coefficients will be biased. One might consider using an IV method to instrument for prices, but we lack data on variables that could serve as clean instruments: those that affect only supply and are unaffected by demand residuals.

## Supporting information

S1 Fig Lorenz curves of organic fruit expenditures across three-person households by Nielsen Scantrack, 2011 through 2013 (December, 2013 $). The dark line in each plot is the actual cumulative household income-household expenditure curve and the lighter line is the 45-degree line. The dashed vertical lines indicate the approximate break point between the middle-class and rich income categories along a market’s annual household income spectrum on the x-axis. Not included on the figure are the breakpoints between the poor and middle-class households in a market, which occur somewhere between 1.1 and 6.2 percent of cumulative income. All household expenditures in year *y* are inflated by households’ year *y* projection factor to arrive at market totals. This figure only includes fruit purchased with a Universal Product Code (UPC).
(TIF)

S2 Fig Lorenz curves of conventional fruit expenditures across two-person households Nielsen Scantrack markets, 2011 through 2013 (December, 2013 $). The dark line in each plot is the actual cumulative household income-household expenditure curve and the lighter line is the 45-degree line. All household expenditures in year *t* are inflated by households’ year *t* projection factor to arrive at market totals. This figure only includes fruit purchased with a Universal Product Code (UPC). In almost every market the poor and middle-class spend more than their share on fruit compared to richer households. It is likely that some of this uneven expenditure patterns across income spectrums are explained by the well-to-do’s tendency to buy more food at restaurants, delis, and farmers’ markets and less on groceries from traditional retail outlets relative to other household types (The JPMorgan Chase Institute 2016). In other words, households from the upper percentiles had less opportunity to buy fruit from stores, typically the place where fruit is sold via UPCs.
(TIF)

S3 Fig Lorenz curves of conventional fruit expenditures across three-person households Nielsen Scantrack markets, 2011 through 2013 (December, 2013 $). The dark line in each plot is the actual cumulative household income-household expenditure curve and the lighter line is the 45-degree line. All household expenditures in year *y* are inflated by households’ year *y* projection factor to arrive at market totals. This figure only includes fruit purchased with a Universal Product Code (UPC). In almost every market the poor and middle-class spend more than their share on fruit compared to richer households. It is likely that some of this uneven expenditure patterns across income spectrums are explained by the well-to-do’s tendency to buy more food at restaurants, delis, and farmers’ markets and less on groceries from traditional retail outlets relative to other household types (The JPMorgan Chase Institute 2016). In other words, households from the upper percentiles had less opportunity to buy fruit from stores, typically the place where fruit is sold via UPCs.
(TIF)

S4 Fig Predicted unconditional and conditional monthly purchase elasticities given a 10% organic fruit price subsidy. Under the subsidy plan all organic fruit prices are 90% of observed 2011–2013 means. Reactions are calculated for a representative lower, middle, and upper income class household. In each plotted-tuple of three points the middle point is the predicted response, the top point is the predicted response plus one standard error, and the bottom point is the predicted response minus one standard error. This figure gives a visual representation of the monthly purchase elasticity predictions from Tables 4 and 5.
(AI) Click here for additional data file.

S5 Fig Predicted unconditional and conditional monthly expenditure elasticities given a 10% organic fruit price subsidy. Under the subsidy plan all organic fruit prices are 90% of observed 2011–2013 means. Reactions are calculated for a representative lower, middle, and upper income class household. In each plotted-tuple of three points the middle point is the predicted response, the top point is the predicted response plus one standard error, and the bottom point is the predicted response minus one standard error. This figure gives a visual representation of the monthly expenditure elasticity predictions from Tables 4 and 5.
(AI) Click here for additional data file.

S1 Table Ounces per fruit item. (DOCX)

S2 Table Real organic expenditures by fruit variety across US households by year (December, 2013 $). Projection factors are used to extrapolate survey level results to national estimates. ‘Other’ expenditure is the sum of expenditures on blackberries, grapes, grapefruit, lemons, raspberries, and miscellaneous.
(DOCX)

S3 Table Average organic fruit price by US region and year (December, 2013 $ per ounce). This data only includes fruit purchased with a Universal Product Code (UPC). Projection factors are used to extrapolate panel level results to national estimates.
(DOCX)

S4 Table Mean household values for each household income class used to predict unconditional demand for each fruit with the econometric estimation methods (separate equations and LinQuad estimation methods). All monetary values are in December, 2013 $.
(DOCX)

S5 Table Mean household values for each household income class used to predict conditional demand for each fruit with the econometric estimation methods (separate equations and LinQuad estimation methods). All monetary values are in December, 2013 $.
(DOCX)

S6 Table Mean household values across each household income class from data used with LASSO estimation method. All monetary values are in December, 2013 $.
(DOCX)

S7 Table The frequency with which a variable’s estimated coefficient is non-zero across 101 estimates of the LASSO model’s selection equation. This table indicates the fraction of 101 LASSO iterations where a variable’s estimated coefficient was non-zero in the selection stage (Eq 9) of the organic fruit consumption model. ‘Dark green indicates that the variable was selected under most or all iterations. Dark red indicates that the variable was selected under few or no iterations. Yellow is the median color on the 0 to 1 scale. ‘HH’ indicates household, ‘H of H’ indicates head of household, ‘RUCC’ indicates the Rural-Urban Continuum Code, and ‘Market’ refers to the 52 Scantrack markets.
(DOCX)

S8 Table The frequency with which a variable’s estimated coefficient is non-zero across 101 estimates of the LASSO model’s quantity equation. This table indicates the fraction of 101 LASSO iterations where a variable’s estimated coefficient was non-zero in the quantity stage (Eq 10) of the organic fruit consumption model. ‘Dark green indicates that the variable was selected under most or all iterations. Dark red indicates that the variable was selected under few or no iterations. Yellow is the median color on the 0 to 1 scale. ‘HH’ indicates household and ‘H of H’ indicates head of household.
(DOCX)

S9 Table The average expected household monthly consumption of organic fruit *i* across all household-months *km*∈*z* where *e*_*ikm*_ > 0 (i.e., conditional demand) when each *km*∈*z*’s expectation is not weighted and is weighted with each *km*∈*z*’s projection factor. (DOCX)

S10 Table The average expected household monthly consumption of organic fruit *i* across all household-months *km*∈*z* (i.e., unconditional demand) when each *km*∈*z*’s expectation is not weighted and is weighted with each *km*∈*z*’s projection factor. (DOCX)

S1 Supporting information Net return to organic farming versus conventional farming. (DOCX)

S2 Supporting information Price imputation. (DOCX)

S3 Supporting information Determining a household’s income class. (DOCX)

S4 Supporting information Data manipulation. (DOCX)

S5 Supporting information Organic fruit demand estimation methods. (DOCX)
