# Correction: The distributional impact of a green payment policy for organic fruit

**DOI:** 10.1371/journal.pone.0215423

**Published:** 2019-04-10

**Authors:** Erik Nelson, John Fitzgerald, Nathan Tefft

The following information is missing from the Data Availability statement: The researchers’ analysis is based on data from the Nielsen Company (US), LLC and marketing databases provided through the Nielsen Datasets at the Kilts Center for Marketing Data Center at The University of Chicago Booth School of Business (https://www.chicagobooth.edu/research/kilts/datasets/nielsen). The authors did not have special access privileges and confirm others are able to access the data in the same manner.

The Competing Interests statement is incorrect. The correct Competing Interests statement is as follows: The conclusions drawn from the Nielsen data are those of the researcher(s) and do not reflect the views of Nielsen. Nielsen is not responsible for, had no role in, and was not involved in analyzing and preparing the results reported herein.
