# Supplementary figures and images for: The distributional impact of a green payment policy for organic fruit
Source: PLoS One. 2019 Feb 7;14(2):e0211199. doi: 10.1371/journal.pone.0211199 (PMC6366746; doi:10.1371/journal.pone.0211199)

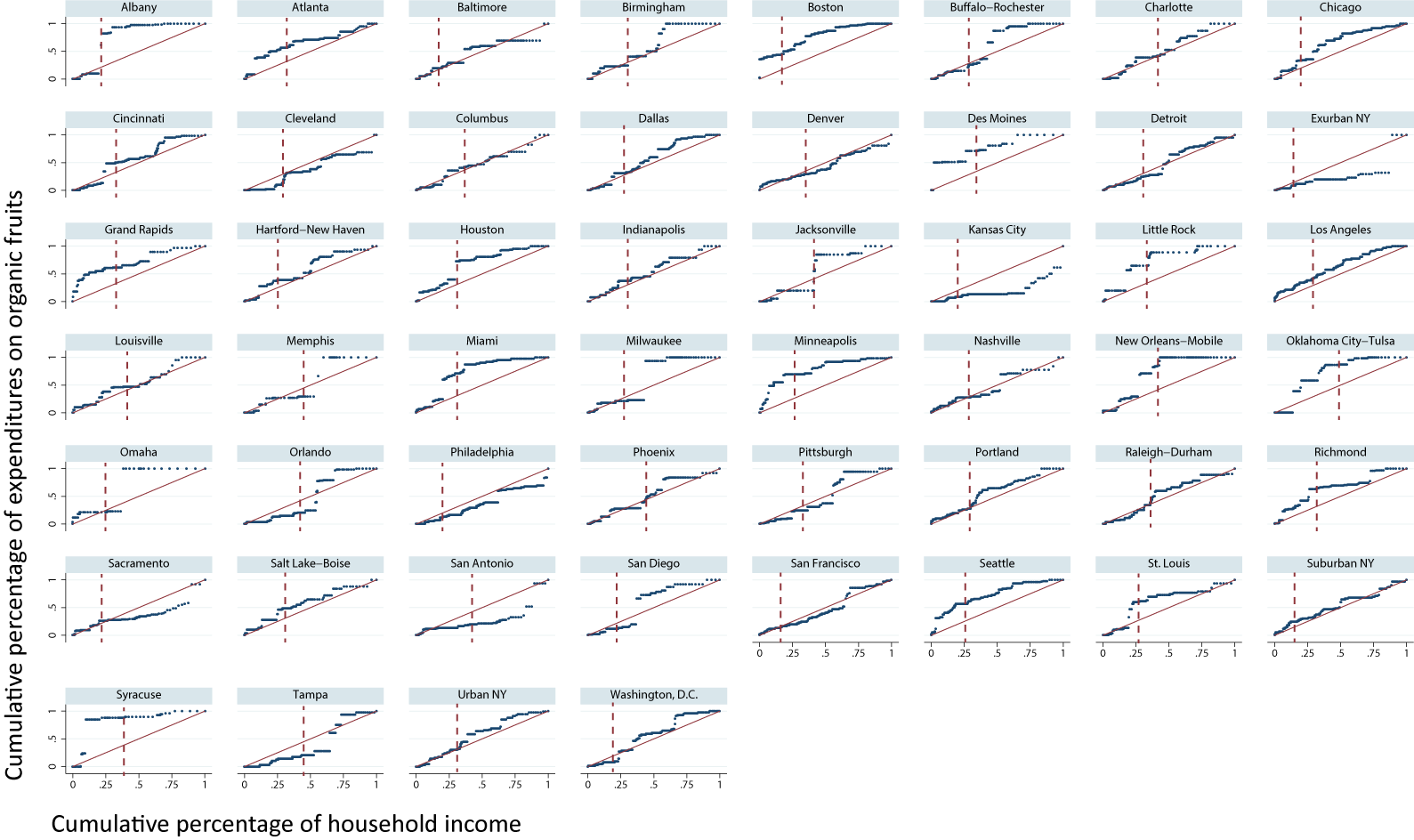

Supplement: S1 Fig — The dark line in each plot is the actual cumulative household income-household expenditure curve and the lighter line is the 45-degree line. The dashed vertical lines indicate the approximate break point between the middle-class and rich income categories along a market’s annual household income spectrum on the x-axis. Not included on the figure are the breakpoints between the poor and middle-class households in a market, which occur somewhere between 1.1 and 6.2 percent of cumulative income. All household expenditures in year y are inflated by households’ year y projection factor to arrive at market totals. This figure only includes fruit purchased with a Universal Product Code (UPC). (TIF) [file pone.0211199.s001.tif]

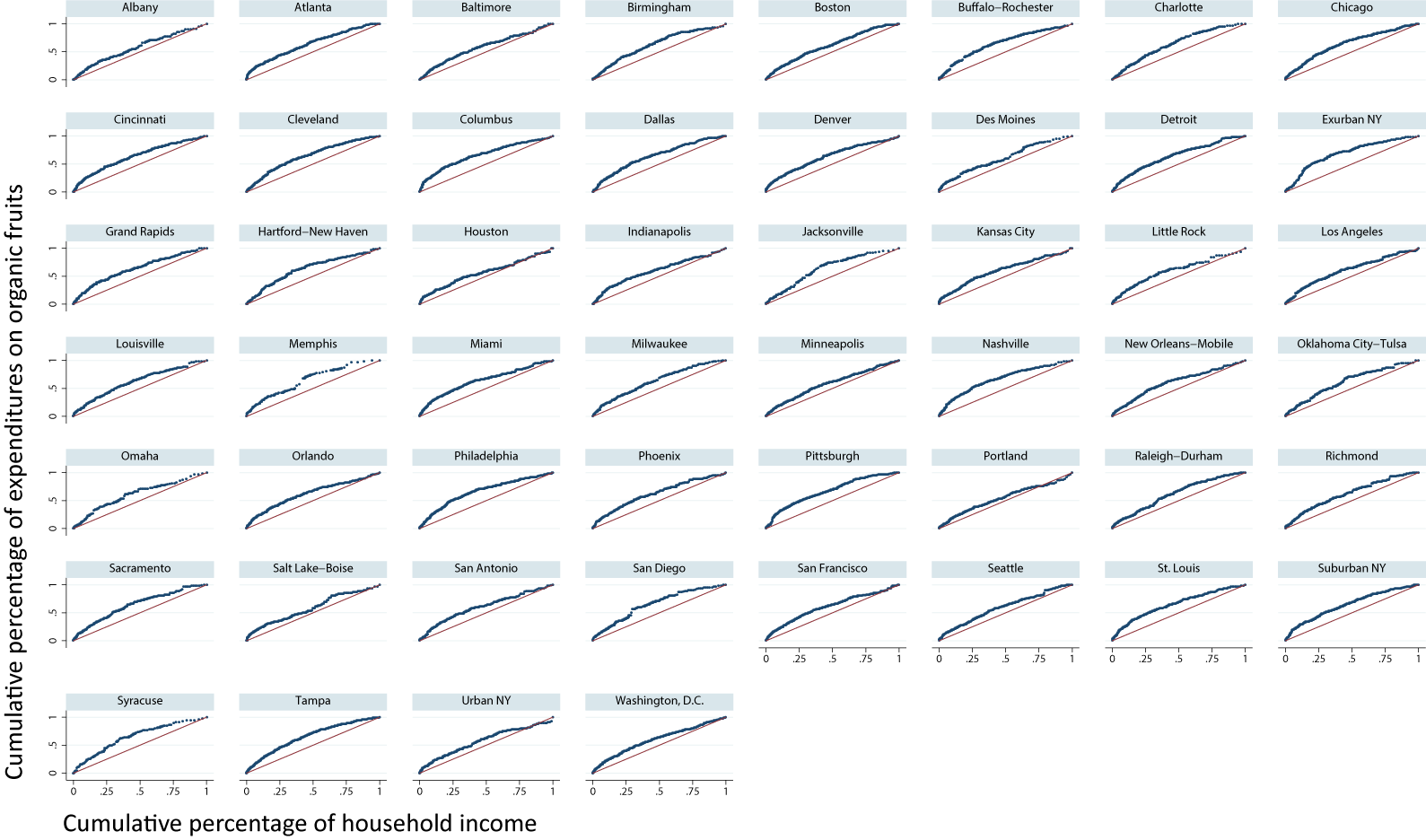

Supplement: S2 Fig — The dark line in each plot is the actual cumulative household income-household expenditure curve and the lighter line is the 45-degree line. All household expenditures in year t are inflated by households’ year t projection factor to arrive at market totals. This figure only includes fruit purchased with a Universal Product Code (UPC). In almost every market the poor and middle-class spend more than their share on fruit compared to richer households. It is likely that some of this uneven expenditure patterns across income spectrums are explained by the well-to-do’s tendency to buy more food at restaurants, delis, and farmers’ markets and less on groceries from traditional retail outlets relative to other household types (The JPMorgan Chase Institute 2016). In other words, households from the upper percentiles had less opportunity to buy fruit from stores, typically the place where fruit is sold via UPCs. (TIF) [file pone.0211199.s002.tif]

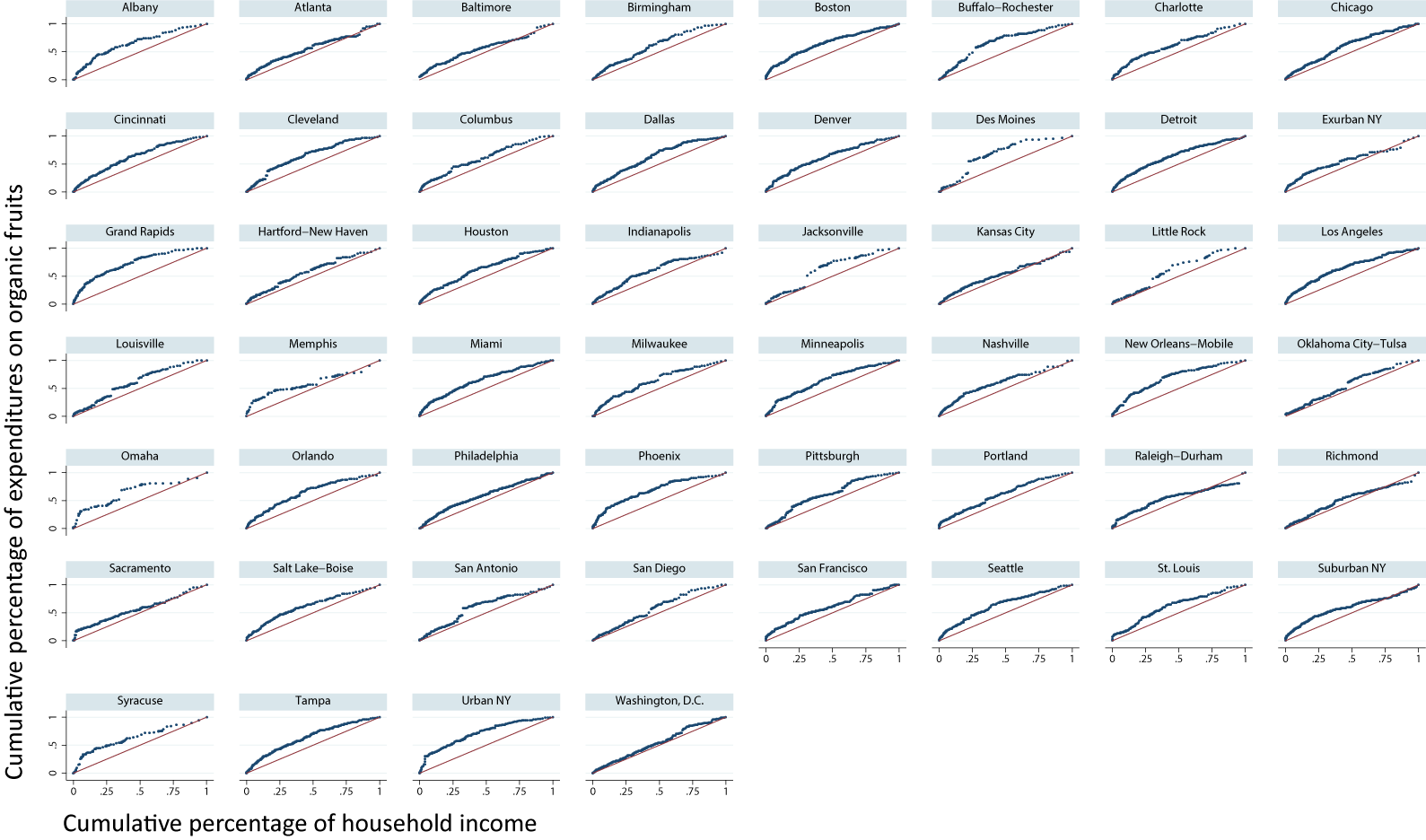

Supplement: S3 Fig — The dark line in each plot is the actual cumulative household income-household expenditure curve and the lighter line is the 45-degree line. All household expenditures in year y are inflated by households’ year y projection factor to arrive at market totals. This figure only includes fruit purchased with a Universal Product Code (UPC). In almost every market the poor and middle-class spend more than their share on fruit compared to richer households. It is likely that some of this uneven expenditure patterns across income spectrums are explained by the well-to-do’s tendency to buy more food at restaurants, delis, and farmers’ markets and less on groceries from traditional retail outlets relative to other household types (The JPMorgan Chase Institute 2016). In other words, households from the upper percentiles had less opportunity to buy fruit from stores, typically the place where fruit is sold via UPCs. (TIF) [file pone.0211199.s003.tif]
