# Supplementary material for: The distributional impact of a green payment policy for organic fruit
Source: PLoS One. 2019 Feb 7;14(2):e0211199. doi: 10.1371/journal.pone.0211199 (PMC6366746; doi:10.1371/journal.pone.0211199)
Supplement: S1 Table — (DOCX) [file pone.0211199.s006.docx]

**S1 Table. Ounces per fruit item**

| **Fruit** | **Ounces** | **Fruit** | **Ounces** |
| --- | --- | --- | --- |
| Apples | 6.420 | Melons | 40.212 |
| Apricots | 1.235 | Mixed | 7.018 |
| Avocados | 7.760 | Nectarine | 5.009 |
| Bananas | 4.162 | Oranges | 4.938 |
| Blackberries | 1.227 | Papayas | 16.861 |
| Blueberries | 27.676 | Passion fruit | 1.499 |
| Cantaloupes | 19.471 | Peaches | 5.291 |
| Cherries | 56.842 | Pears | 6.279 |
| Citrine | 3.104 | Persimmon | 5.926 |
| Coconuts | 14.004 | Pineapples | 31.923 |
| Dragonfruit | 13.228 | Plantains | 6.314 |
| Figs | 1.764 | Plums | 2.328 |
| Goldenberry | 24.594 | Pomegranates | 9.947 |
| Grapefruits | 6.226 | Pummelos | 21.482 |
| Grapes | 24.594 | Quince | 6.279 |
| Honeydew | 35.274 | Raspberry | 1.227 |
| Kiwis | 2.434 | Starfruit | 3.210 |
| Lemons | 2.504 | Strawberries | 10.785 |
| Limes | 2.363 | Tangelos | 3.104 |
| Lychee | 0.705 | Tangerines | 3.104 |
| Mandarin | 3.104 | Ugli fruit | 3.104 |
| Mangos | 11.852 | Watermelons | 159.368 |
