# Supplementary material for: The distributional impact of a green payment policy for organic fruit
Source: PLoS One. 2019 Feb 7;14(2):e0211199. doi: 10.1371/journal.pone.0211199 (PMC6366746; doi:10.1371/journal.pone.0211199)
Supplement: S2 Table — Projection factors are used to extrapolate survey level results to national estimates. ‘Other’ expenditure is the sum of expenditures on blackberries, grapes, grapefruit, lemons, raspberries, and miscellaneous. (DOCX) [file pone.0211199.s007.docx]

**S2 Table: Real organic expenditures by fruit variety across US households by year (December, 2013 $).**

|  | **2011** | **2012** | **2013** |
| --- | --- | --- | --- |
| **Apples** | 28.80 | 33.20 | 43.80 |
| **Blueberries** | 19.70 | 27.10 | 38.90 |
| **Oranges** | 7.16 | 7.48 | 8.42 |
| **Strawberries** | 47.90 | 53.70 | 58.00 |
| **Other** | 41.20 | 46.50 | 62.40 |
| Blackberries | 8.86 | 6.86 | 9.32 |
| Grapes | 2.48 | 5.10 | 8.70 |
| Grapefruit | 1.32 | 1.07 | 1.23 |
| Lemons | 2.51 | 2.30 | 3.48 |
| Raspberries | 19.90 | 22.20 | 21.30 |
| Misc. | 6.14 | 8.96 | 18.40 |
| **Total** | 144.76 | 167.98 | 211.52 |
