# Supplementary material for: The distributional impact of a green payment policy for organic fruit
Source: PLoS One. 2019 Feb 7;14(2):e0211199. doi: 10.1371/journal.pone.0211199 (PMC6366746; doi:10.1371/journal.pone.0211199)
Supplement: S3 Table — This data only includes fruit purchased with a Universal Product Code (UPC). Projection factors are used to extrapolate panel level results to national estimates. (DOCX) [file pone.0211199.s008.docx]

**S3 Table: Average organic fruit price by US region and year (Dec, 2013 $ per ounce).**

|  |  | **Census Region** | | | | | | | | | |
| --- | --- | --- | --- | --- | --- | --- | --- | --- | --- | --- | --- |
|  | **Year** | **1** | **2** | **3** | **4** | **5** | **6** | **7** | **8** | **9** | **National** |
| **Apple** | **2011** | 0.100 | 0.089 | 0.086 | 0.085 | 0.098 | 0.085 | 0.087 | 0.084 | 0.081 | 0.088 |
|  | **2012** | 0.101 | 0.107 | 0.095 | 0.091 | 0.099 | 0.096 | 0.090 | 0.097 | 0.096 | 0.097 |
|  | **2013** | 0.114 | 0.109 | 0.100 | 0.092 | 0.108 | 0.101 | 0.089 | 0.090 | 0.088 | 0.099 |
| **Blueberries** | **2011** | 0.554 | 0.477 | 0.415 | 0.477 | 0.443 | 0.484 | 0.505 | 0.486 | 0.500 | 0.472 |
|  | **2012** | 0.583 | 0.563 | 0.464 | 0.461 | 0.543 | 0.509 | 0.519 | 0.549 | 0.543 | 0.525 |
|  | **2013** | 0.477 | 0.522 | 0.491 | 0.481 | 0.488 | 0.534 | 0.494 | 0.490 | 0.519 | 0.500 |
| **Oranges** | **2011** | 0.091 | 0.091 | 0.076 | 0.082 | 0.088 | 0.077 | 0.088 | 0.085 | 0.080 | 0.084 |
|  | **2012** | 0.093 | 0.095 | 0.076 | 0.084 | 0.086 | 0.083 | 0.085 | 0.082 | 0.082 | 0.085 |
|  | **2013** | 0.094 | 0.091 | 0.074 | 0.089 | 0.090 | 0.081 | 0.091 | 0.078 | 0.083 | 0.085 |
| **Strawberries** | **2011** | 0.281 | 0.291 | 0.254 | 0.274 | 0.283 | 0.244 | 0.281 | 0.255 | 0.256 | 0.270 |
|  | **2012** | 0.279 | 0.282 | 0.242 | 0.262 | 0.273 | 0.243 | 0.266 | 0.232 | 0.230 | 0.257 |
|  | **2013** | 0.282 | 0.291 | 0.259 | 0.270 | 0.291 | 0.278 | 0.271 | 0.239 | 0.259 | 0.272 |
