# Supplementary material for: The distributional impact of a green payment policy for organic fruit
Source: PLoS One. 2019 Feb 7;14(2):e0211199. doi: 10.1371/journal.pone.0211199 (PMC6366746; doi:10.1371/journal.pone.0211199)
Supplement: S4 Table — All monetary values are in December, 2013 $. (DOCX) [file pone.0211199.s009.docx]

**S4 Table: Mean household values for each income class used to predict unconditional demand for each fruit with the econometric estimation methods (separate equations and LinQuad estimation methods).**

| **Variable** | **Poor** | **Middle** | **Rich** |
| --- | --- | --- | --- |
| Monthly Income | 1,254 | 3,839 | 9,206 |
| At least one child in the household (fraction) | 0.283 | 0.237 | 0.184 |
| Male or female head of household has college degree (fraction) | 0.316 | 0.465 | 0.741 |
| Live in a metropolitan area (fraction) | 0.763 | 0.820 | 0.904 |
| Household heads are married (fraction) | 0.431 | 0.649 | 0.734 |
| Household identifies as black (fraction) | 0.105 | 0.088 | 0.099 |
| Household identifies as Asian (fraction) | 0.017 | 0.023 | 0.047 |
| Household identifies as other (fraction) | 0.057 | 0.042 | 0.037 |
| Household Size | 2.525 | 2.454 | 2.313 |
| Average price of organic apples ($ per ounce) | 0.094 | 0.094 | 0.095 |
| Average price of conventional apples ($ per ounce) | 0.075 | 0.075 | 0.076 |
| Average price of organic blueberries ($ per ounce) | 0.491 | 0.492 | 0.499 |
| Average price of conventional blueberries ($ per ounce) | 0.314 | 0.315 | 0.321 |
| Average price of organic oranges ($ per ounce) | 0.083 | 0.084 | 0.085 |
| Average price of conventional oranges ($ per ounce) | 0.064 | 0.064 | 0.064 |
| Average price of organic strawberries ($ per ounce) | 0.265 | 0.266 | 0.267 |
| Average price of conventional strawberries ($ per ounce) | 0.149 | 0.149 | 0.151 |
| Average price of other organic fruits ($ per ounce) | 0.332 | 0.333 | 0.335 |
| Average price of other conventional fruits ($ per ounce) | 0.153 | 0.153 | 0.155 |
