# Supplementary material for: The distributional impact of a green payment policy for organic fruit
Source: PLoS One. 2019 Feb 7;14(2):e0211199. doi: 10.1371/journal.pone.0211199 (PMC6366746; doi:10.1371/journal.pone.0211199)
Supplement: S5 Table — All monetary values are in December, 2013 $. (DOCX) [file pone.0211199.s010.docx]

**S5 Table. Mean household values for each household income class used to predict conditional demand for each fruit with the econometric estimation methods (separate equations and LinQuad estimation methods).**

|  | **Apples** | | | **Blueberries** | | | **Oranges** | | | **Strawberries** | | |
| --- | --- | --- | --- | --- | --- | --- | --- | --- | --- | --- | --- | --- |
| **Variable** | **Poor** | **Middle** | **Rich** | **Poor** | **Middle** | **Rich** | **Poor** | **Middle** | **Rich** | **Poor** | **Middle** | **Rich** |
| Monthly Income | 1,444 | 4,338 | 9,970 | 1,075 | 4,082 | 9,923 | 1,344 | 3,991 | 9,449 | 1,187 | 4,204 | 9,989 |
| At least one child in the household (fraction) | 0.455 | 0.393 | 0.317 | 0.293 | 0.284 | 0.207 | 0.340 | 0.268 | 0.235 | 0.332 | 0.333 | 0.275 |
| Male or female head of household has college degree (fraction) | 0.514 | 0.604 | 0.824 | 0.511 | 0.626 | 0.846 | 0.392 | 0.501 | 0.784 | 0.498 | 0.627 | 0.847 |
| Live in a metropolitan area (fraction) | 0.798 | 0.864 | 0.946 | 0.901 | 0.908 | 0.946 | 0.725 | 0.806 | 0.889 | 0.847 | 0.906 | 0.943 |
| Household heads are married (fraction) | 0.581 | 0.748 | 0.807 | 0.382 | 0.639 | 0.752 | 0.497 | 0.713 | 0.749 | 0.425 | 0.663 | 0.770 |
| Household identifies as black (fraction) | 0.079 | 0.051 | 0.096 | 0.040 | 0.051 | 0.073 | 0.052 | 0.072 | 0.112 | 0.064 | 0.054 | 0.068 |
| Household identifies as Asian (fraction) | 0.041 | 0.049 | 0.086 | 0.045 | 0.048 | 0.072 | 0.020 | 0.023 | 0.038 | 0.028 | 0.043 | 0.068 |
| Household identifies as other (fraction) | 0.068 | 0.064 | 0.040 | 0.097 | 0.042 | 0.038 | 0.111 | 0.043 | 0.049 | 0.091 | 0.044 | 0.037 |
| Household Size | 3.103 | 2.892 | 2.624 | 2.335 | 2.451 | 2.322 | 2.725 | 2.654 | 2.393 | 2.454 | 2.580 | 2.436 |
| Average price of organic apples ($ per ounce) | 0.096 | 0.097 | 0.097 | 0.091 | 0.093 | 0.092 | 0.095 | 0.094 | 0.094 | 0.092 | 0.091 | 0.093 |
| Average price of conventional apples ($ per ounce) | 0.078 | 0.078 | 0.078 | 0.077 | 0.077 | 0.077 | 0.076 | 0.078 | 0.079 | 0.078 | 0.077 | 0.078 |
| Average price of organic blueberries ($ per ounce) | 0.514 | 0.509 | 0.522 | 0.461 | 0.444 | 0.468 | 0.476 | 0.474 | 0.478 | 0.480 | 0.476 | 0.482 |
| Average price of conventional blueberries ($ per ounce) | 0.351 | 0.346 | 0.355 | 0.287 | 0.291 | 0.304 | 0.290 | 0.305 | 0.311 | 0.316 | 0.304 | 0.316 |
| Average price of organic oranges ($ per ounce) | 0.084 | 0.086 | 0.086 | 0.079 | 0.080 | 0.081 | 0.080 | 0.081 | 0.083 | 0.081 | 0.080 | 0.081 |
| Average price of conventional oranges ($ per ounce) | 0.062 | 0.062 | 0.062 | 0.061 | 0.060 | 0.061 | 0.061 | 0.060 | 0.063 | 0.067 | 0.066 | 0.067 |
| Average price of organic strawberries ($ per ounce) | 0.275 | 0.273 | 0.275 | 0.262 | 0.264 | 0.265 | 0.267 | 0.264 | 0.267 | 0.237 | 0.234 | 0.243 |
| Average price of conventional strawberries ($ per ounce) | 0.152 | 0.157 | 0.157 | 0.147 | 0.149 | 0.149 | 0.147 | 0.146 | 0.151 | 0.140 | 0.139 | 0.142 |
| Average price of other organic fruits ($ per ounce) | 0.332 | 0.339 | 0.345 | 0.323 | 0.326 | 0.329 | 0.332 | 0.337 | 0.331 | 0.326 | 0.321 | 0.326 |
| Average price of other conventional fruits ($ per ounce) | 0.158 | 0.160 | 0.162 | 0.159 | 0.161 | 0.163 | 0.165 | 0.159 | 0.162 | 0.158 | 0.156 | 0.160 |
