# Supplementary material for: The distributional impact of a green payment policy for organic fruit
Source: PLoS One. 2019 Feb 7;14(2):e0211199. doi: 10.1371/journal.pone.0211199 (PMC6366746; doi:10.1371/journal.pone.0211199)
Supplement: S6 Table — All monetary values are in December, 2013 $. (DOCX) [file pone.0211199.s011.docx]

**S6 Table. Mean household values across each household income class from data used with LASSO estimation methods.**

|  | **Income class** | | |
| --- | --- | --- | --- |
| **Variable** | **Poor** | **Middle** | **Rich** |
| Monthly Income | 1,234 | 3,868 | 9,260 |
| At least one child in the household (fraction) | 0.271 | 0.238 | 0.186 |
| Male or female head of household has college degree (fraction) | 0.318 | 0.466 | 0.747 |
| Live in a metropolitan area (fraction) | 0.841 | 0.879 | 0.937 |
| Household heads are married (fraction) | 0.420 | 0.635 | 0.722 |
| Household identifies as black (fraction) | 0.115 | 0.097 | 0.107 |
| Household identifies as Asian (fraction) | 0.020 | 0.026 | 0.051 |
| Household identifies as other (fraction) | 0.057 | 0.044 | 0.037 |
| Household Size | 2.487 | 2.456 | 2.314 |
| Average price of organic apples ($ per ounce) | 0.094 | 0.094 | 0.094 |
| Average price of conventional apples ($ per ounce) | 0.075 | 0.075 | 0.076 |
| Average price of organic blueberries ($ per ounce) | 0.495 | 0.495 | 0.503 |
| Average price of conventional blueberries ($ per ounce) | 0.316 | 0.317 | 0.323 |
| Average price of organic oranges ($ per ounce) | 0.083 | 0.084 | 0.085 |
| Average price of conventional oranges ($ per ounce) | 0.064 | 0.064 | 0.065 |
| Average price of organic strawberries ($ per ounce) | 0.268 | 0.268 | 0.269 |
| Average price of conventional strawberries ($ per ounce) | 0.149 | 0.150 | 0.151 |
| Average price of other organic fruits ($ per ounce) | 0.352 | 0.316 | 0.344 |
| Average price of other conventional fruits ($ per ounce) | 0.140 | 0.146 | 0.168 |
