# Supplementary material for: The distributional impact of a green payment policy for organic fruit
Source: PLoS One. 2019 Feb 7;14(2):e0211199. doi: 10.1371/journal.pone.0211199 (PMC6366746; doi:10.1371/journal.pone.0211199)
Supplement: S7 Table — This table indicates the fraction of 101 LASSO iterations where a variable’s estimated coefficient was non-zero in the selection stage (Eq 9) of the organic fruit consumption model. ‘Dark green indicates that the variable was selected under most or all iterations. Dark red indicates that the variable was selected under few or no iterations. Yellow is the median color on the 0 to 1 scale. ‘HH’ indicates household, ‘H of H’ indicates head of household, ‘RUCC’ indicates the Rural-Urban Continuum Code, and ‘Market’ refers to the 52 Scantrack markets. (DOCX) [file pone.0211199.s012.docx]

**S7 Table. The frequency with which a variable’s estimated coefficient is non-zero across 101 estimates of the LASSO model’s selection equation.**

|  | **Apple** | | | **Blueberry** | | | **Orange** | | | **Strawberry** | | |
| --- | --- | --- | --- | --- | --- | --- | --- | --- | --- | --- | --- | --- |
| **Income class** | **Poor** | **Middle** | **Rich** | **Poor** | **Middle** | **Rich** | **Poor** | **Middle** | **Rich** | **Poor** | **Middle** | **Rich** |
| **Price variables** |  |  |  |  |  |  |  |  |  |  |  |  |
| Organic apple | 0.72 | 0.98 | 1.00 | 0.89 | 0.85 | 0.96 | 0.67 | 0.56 | 0.66 | 0.93 | 0.92 | 0.79 |
| Conventional apple | 1.00 | 0.98 | 1.00 | 0.94 | 1.00 | 1.00 | 0.85 | 1.00 | 1.00 | 0.98 | 1.00 | 1.00 |
| Organic blueberries | 0.80 | 0.72 | 0.98 | 0.91 | 0.98 | 0.84 | 0.78 | 0.53 | 0.61 | 0.69 | 0.78 | 0.98 |
| Conventional blueberries | 0.91 | 0.98 | 0.99 | 0.77 | 0.79 | 0.99 | 0.71 | 0.41 | 0.76 | 0.99 | 1.00 | 1.00 |
| Organic oranges | 0.69 | 0.72 | 0.64 | 0.76 | 1.00 | 1.00 | 0.74 | 0.87 | 0.83 | 0.99 | 0.99 | 1.00 |
| Conventional oranges | 0.40 | 0.68 | 0.81 | 0.62 | 1.00 | 1.00 | 0.70 | 0.98 | 0.66 | 0.98 | 0.76 | 0.99 |
| Organic strawberries | 0.89 | 0.81 | 0.87 | 0.92 | 1.00 | 0.68 | 0.68 | 0.57 | 0.73 | 1.00 | 1.00 | 1.00 |
| Conventional strawberries | 0.74 | 0.97 | 0.96 | 0.93 | 1.00 | 1.00 | 0.70 | 0.62 | 0.83 | 1.00 | 1.00 | 1.00 |
| Other organics | 0.54 | 0.95 | 0.98 | 0.73 | 1.00 | 1.00 | 0.71 | 0.83 | 0.68 | 0.66 | 0.98 | 1.00 |
| Other conventional | 0.81 | 0.98 | 1.00 | 0.98 | 1.00 | 1.00 | 0.92 | 1.00 | 1.00 | 0.98 | 1.00 | 1.00 |
| **Socio – economic variables** |  |  |  |  |  |  |  |  |  |  |  |  |
| HH income | 0.50 | 0.98 | 0.92 | 0.99 | 1.00 | 1.00 | 0.84 | 0.65 | 0.79 | 0.61 | 1.00 | 1.00 |
| Children | 0.69 | 0.88 | 0.84 | 0.86 | 0.85 | 0.84 | 0.78 | 0.72 | 0.81 | 0.78 | 0.96 | 0.92 |
| Marital status | 0.54 | 0.77 | 0.76 | 0.71 | 0.80 | 0.68 | 0.72 | 0.72 | 0.76 | 0.77 | 0.74 | 0.74 |
| HH composition | 0.54 | 0.66 | 0.59 | 0.65 | 0.67 | 0.76 | 0.69 | 0.53 | 0.67 | 0.73 | 0.83 | 0.82 |
| HH size | 0.61 | 0.76 | 0.82 | 0.50 | 0.81 | 0.89 | 0.63 | 0.68 | 0.75 | 0.68 | 0.88 | 0.86 |
| Residential type | 0.67 | 0.82 | 0.91 | 0.82 | 0.85 | 0.93 | 0.78 | 0.73 | 0.75 | 0.88 | 0.91 | 0.94 |
| Race | 0.68 | 0.91 | 0.81 | 0.90 | 0.90 | 0.89 | 0.84 | 0.76 | 0.78 | 0.82 | 0.98 | 0.90 |
| Hispanic | 0.61 | 0.76 | 0.97 | 0.94 | 0.86 | 0.77 | 0.71 | 0.66 | 0.97 | 0.89 | 0.81 | 0.85 |
| H of H male age | 0.66 | 0.69 | 0.75 | 0.72 | 0.71 | 0.72 | 0.69 | 0.69 | 0.66 | 0.71 | 0.82 | 0.76 |
| H of H female age | 0.65 | 0.72 | 0.75 | 0.72 | 0.74 | 0.73 | 0.70 | 0.62 | 0.69 | 0.74 | 0.80 | 0.80 |
| H of H male education | 0.79 | 0.77 | 0.71 | 0.83 | 0.80 | 0.77 | 0.71 | 0.65 | 0.69 | 0.78 | 0.80 | 0.80 |
| H of H female education | 0.73 | 0.70 | 0.77 | 0.77 | 0.90 | 0.86 | 0.66 | 0.66 | 0.65 | 0.81 | 0.79 | 0.90 |
| H of H male hours worked | 0.52 | 0.71 | 0.55 | 0.63 | 0.61 | 0.65 | 0.54 | 0.63 | 0.50 | 0.60 | 0.56 | 0.67 |
| H of H female hours worked | 0.57 | 0.70 | 0.58 | 0.48 | 0.59 | 0.50 | 0.57 | 0.53 | 0.61 | 0.58 | 0.69 | 0.57 |
| H of H male occupation | 0.70 | 0.81 | 0.76 | 0.75 | 0.78 | 0.77 | 0.73 | 0.74 | 0.72 | 0.88 | 0.87 | 0.86 |
| H of H female occupation | 0.62 | 0.78 | 0.75 | 0.63 | 0.74 | 0.81 | 0.63 | 0.65 | 0.75 | 0.60 | 0.79 | 0.88 |
| **Misc. variables** |  |  |  |  |  |  |  |  |  |  |  |  |
| Market | 0.71 | 0.84 | 0.83 | 0.85 | 0.87 | 0.89 | 0.74 | 0.78 | 0.86 | 0.87 | 0.94 | 0.92 |
| Rural-Urban Continuum Codes | 0.74 | 0.83 | 0.89 | 0.78 | 0.96 | 0.87 | 0.85 | 0.76 | 0.84 | 0.81 | 0.93 | 0.93 |
| Season | 0.55 | 0.78 | 0.71 | 0.72 | 0.73 | 0.68 | 0.63 | 0.64 | 0.79 | 0.71 | 0.86 | 0.89 |
