# Supplementary material for: The distributional impact of a green payment policy for organic fruit
Source: PLoS One. 2019 Feb 7;14(2):e0211199. doi: 10.1371/journal.pone.0211199 (PMC6366746; doi:10.1371/journal.pone.0211199)
Supplement: S9 Table — (DOCX) [file pone.0211199.s014.docx]

**S9 Table. The average expected household monthly consumption of organic fruit *i* across all household-months *km***$\boldsymbol{\in}$***z* where *e_ikm_* > 0 (i.e., conditional demand) when each *km***$\boldsymbol{\in}$***z*’s expectation is not weighted and is weighted with each *km***$\boldsymbol{\in}$***z*’s projection factor.**

|  | **Unweighted mean purchased (Oz per month)** | | | **Weighted mean purchased (Oz per month)** | | | **Weighted mean / Unweighted mean** | | |
| --- | --- | --- | --- | --- | --- | --- | --- | --- | --- |
|  | Income class | | | Income class | | | Income class | | |
|  | Low | Middle | High | Low | Middle | High | Low | Middle | High |
| **Est. method** | **Apples** | | | | | | | | |
| Separate equations | 73.95 | 83.33 | 77.70 | 69.80 | 85.31 | 80.88 | 0.944 | 1.024 | 1.041 |
| LinQuad | 77.39 | 88.98 | 78.33 | 65.45 | 88.84 | 80.53 | 0.846 | 0.998 | 1.028 |
| LASSO | 82.98 | 88.62 | 81.81 | 82.98 | 87.47 | 83.20 | 1.000 | 0.987 | 1.017 |
|  | **Blueberries** | | | | | | | | |
| Separate equations | 18.28 | 17.07 | 17.21 | 16.70 | 16.18 | 17.42 | 0.914 | 0.948 | 1.012 |
| LinQuad | 17.22 | 17.33 | 17.99 | 16.63 | 15.15 | 18.24 | 0.966 | 0.874 | 1.014 |
| LASSO | 18.35 | 17.04 | 16.95 | 15.52 | 15.97 | 17.23 | 0.846 | 0.937 | 1.016 |
|  | **Oranges** | | | | | | | | |
| Separate equations | 69.48 | 71.96 | 70.00 | 71.96 | 70.18 | 69.63 | 1.036 | 0.975 | 0.995 |
| LinQuad | 82.71 | 72.92 | 77.28 | 93.79 | 65.43 | 74.57 | 1.134 | 0.897 | 0.965 |
| LASSO | 68.40 | 74.13 | 70.87 | 68.40 | 74.13 | 72.74 | 1.000 | 1.000 | 1.026 |
|  | **Strawberries** | | | | | | | | |
| Separate equations | 25.34 | 25.75 | 26.70 | 24.72 | 25.78 | 26.97 | 0.976 | 1.001 | 1.010 |
| LinQuad | 26.08 | 25.86 | 26.90 | 24.16 | 25.02 | 27.13 | 0.926 | 0.968 | 1.008 |
| LASSO | 25.43 | 26.58 | 26.41 | 25.43 | 26.00 | 26.10 | 1.000 | 0.978 | 1.011 |
