# Supplementary material for: The distributional impact of a green payment policy for organic fruit
Source: PLoS One. 2019 Feb 7;14(2):e0211199. doi: 10.1371/journal.pone.0211199 (PMC6366746; doi:10.1371/journal.pone.0211199)
Supplement: S10 Table — (DOCX) [file pone.0211199.s015.docx]

**S10 Table. The average expected household monthly consumption of organic fruit *i* across all household-months *km***$\boldsymbol{\in}$***z* (i.e., unconditional demand) when each *km***$\boldsymbol{\in}$***z*’s expectation is not weighted and is weighted with each *km***$\boldsymbol{\in}$***z*’s projection factor.**

|  | **Unweighted mean purchased (Oz per month)** | | | **Weighted mean purchased (Oz per month)** | | | **Weighted mean / Unweighted mean** | | |
| --- | --- | --- | --- | --- | --- | --- | --- | --- | --- |
|  | Income class | | | Income class | | | Income class | | |
|  | Low | Middle | High | Low | Middle | High | Low | Middle | High |
| **Est. method** | **Apples** | | | | | | | | |
| Separate equations | 0.570 | 0.644 | 0.782 | 0.452 | 0.641 | 0.863 | 0.793 | 0.994 | 1.105 |
| LinQuad | 0.243 | 0.360 | 0.434 | 0.183 | 0.347 | 0.473 | 0.753 | 0.964 | 1.090 |
| LASSO | 1.502 | 0.957 | 1.005 | 1.270 | 0.818 | 1.209 | 0.846 | 0.855 | 1.203 |
|  | **Blueberries** | | | | | | | | |
| Separate equations | 0.183 | 0.125 | 0.254 | 0.153 | 0.110 | 0.272 | 0.838 | 0.875 | 1.072 |
| LinQuad | 0.043 | 0.050 | 0.116 | 0.040 | 0.044 | 0.117 | 0.930 | 0.880 | 1.009 |
| LASSO | 0.270 | 0.131 | 0.243 | 0.171 | 0.111 | 0.251 | 0.633 | 0.847 | 1.033 |
|  | **Oranges** | | | | | | | | |
| Separate equations | 0.228 | 0.164 | 0.193 | 0.221 | 0.141 | 0.179 | 0.966 | 0.864 | 0.924 |
| LinQuad | 0.078 | 0.079 | 0.106 | 0.085 | 0.067 | 0.103 | 1.090 | 0.848 | 0.972 |
| LASSO | 0.450 | 0.161 | 0.200 | 0.369 | 0.151 | 0.203 | 0.820 | 0.938 | 1.015 |
|  | **Strawberries** | | | | | | | | |
| Separate equations | 0.401 | 0.419 | 0.699 | 0.354 | 0.416 | 0.724 | 0.882 | 0.992 | 1.035 |
| LinQuad | 0.127 | 0.150 | 0.314 | 0.109 | 0.140 | 0.323 | 0.858 | 0.933 | 1.029 |
| LASSO | 0.800 | 0.596 | 0.765 | 0.467 | 0.583 | 0.809 | 0.584 | 0.978 | 1.058 |
