# Supplementary material for: The distributional impact of a green payment policy for organic fruit
Source: PLoS One. 2019 Feb 7;14(2):e0211199. doi: 10.1371/journal.pone.0211199 (PMC6366746; doi:10.1371/journal.pone.0211199)
Supplement: S1 Supporting information — (DOCX) [file pone.0211199.s016.docx]

**S1 Supporting information. Net returns to organic farming versus conventional farming.**

Evidence suggests that subsidization of organic food production would likely be welfare creating even if the external benefits were small. In a survey of 44 studies, representing 55 crops grown in 14 countries on five continents, Crowder and Reganold (2015) found that, on average, the net present value of organic crop production was 35 percent greater than the net present value from conventional crop production. In the US organic corn-soybean rotation systems have proven to be more profitable than their conventional analogs (Delbridge et al. 2011). In many cases organic food price premiums more than cover their additional cost. This begs the question: if organic agriculture production generates premiums more than cover their additional cost.

This begs the question: if organic agriculture production generates larger net returns than conventional agriculture why is it not more prevalent on the US landscape? US farmers have identified several barriers to entering the organic production market, including the large upfront cost of transitioning to organic farming, the bureaucratic process of certification, having to learn and experiment with new production techniques, weak organic farming and marketing infrastructure, and a federal subsidy system focused on conventional farming (Delbridge and King 2016, Delbridge et al. 2017).

Crowder, D.W. and J.P. Reganold. “Financial competitiveness of organic agriculture on a global scale.” *Proceedings of the National Academy of Sciences*, 112(24), 2015, 7611-7616.

Delbridge, T. A., J. A. Coulter, R. P. King, C. C. Sheaffer, and D. L. Wyse. “Economic Performance of Long-Term Organic and Conventional Cropping Systems in Minnesota.” *Agronomy Journal*, 103, 2011, 1372-1382.

Delbridge, Timothy A., and Robert P. King. 2016. Transitioning to Organic Crop Production: A Dynamic Programming Approach. *Journal of Agricultural & Resource Economics* 41.3, 2016.

Delbridge, T.A., R.P. King, G. Short., and K. James. "Risk and Red Tape: Barriers to Organic Transition for U.S. Farmers." *Choices*, Quarter 4, 2017. Available online: http://www.choicesmagazine.org/choices-magazine/submitted-articles/risk-and-red-tape-barriers-to-organic-transition-for-us-farmers.
