# Supplementary material for: The distributional impact of a green payment policy for organic fruit
Source: PLoS One. 2019 Feb 7;14(2):e0211199. doi: 10.1371/journal.pone.0211199 (PMC6366746; doi:10.1371/journal.pone.0211199)
Supplement: S2 Supporting information — (DOCX) [file pone.0211199.s017.docx]

**S2 Supporting information. Price imputation.**

Using the raw Nielsen data on household expenditures and purchases on all consumable items, we first created household-month expenditure (represented with *e* and measured in dollars) and purchase (represented with *o* and measured in ounces) variables for 20 fruit type $\times$ varieties,

$e_{fkm}=\sum_{d\in m} e_{fkd}$ (A)

$o_{fkm}=\sum_{d\in m} o_{fkd}$ (B)

where *f* indexes fruit type $\times$ variety*, d* indexes each shopping trip taken that ended up in a fruit purchase, and *m* indexes months in the years 2011, 2012, and 2013. In some cases purchases were reported in the number of items purchased, not ounces purchased. In these cases we had to first convert number of items purchased to ounces purchased before we could add the purchase to other purchases. The weight per item of fruit is given in Table B.

Note that *e_fkm_* and *o_fkm_* for two fruit type $\times$ varieties, ‘other $\times$ organic’ and ‘other $\times$ conventional’, are created by summing *km*’s monthly expenditures and purchases of minor organic fruit types and minor conventional fruit types, respectively. See section XX of the paper for more details.

Then we calculated the nominal price per ounce of *f* faced by household *k* in month *m* for each unique *fkm* combination,

*P_fkm_* = *e_fkm_* / *o_fkm_* (C)

When $e_{fkm}$ and $o_{fkm}$ were 0 (household *k* did not purchase *f* during the month *m*) we had to impute *P_fkm_*.

We created two sets of imputed prices. The first set of imputed prices were created with the following method. Let, $\hat{P}_{fmry}$ be the imputed price of fruit type $\times$ variety *f* in month *m* in market *r* in year *y*,

$\hat{P}_{fmry}=\frac{\frac{\sum_{km\in y} \left[ w_{ky}I\left( k\in r,y \right)e_{fkm} \right]}{\sum_{km\in y} \left[ w_{ky}I\left( k\in r,y \right)I\left( e_{fkm}>0 \right) \right]}}{\frac{\sum_{km} w_{km}I\left( km\in r \right)o_{fkm}}{\sum_{km} w_{km}I\left( km\in r \right)I\left( e_{fkm}>0 \right)}}$ (D)

where $km\in y$ is the set of all *km* observations in year *y, w_ky_* is household *k*’s projection factor in year *y*, $I\left( k\in r,y \right)=1$ if household *k* resides in market *r* in year *y* and equals 0 otherwise, and $I\left( e_{fkm}>0 \right)=1$ if $e_{fkm}>0$ and equals 0 otherwise. Therefore, $\hat{P}_{fmry}$ is the (weighted) average price of *f* across all household-month purchases of *f* in month *m* of year *y* in market *r*. When $\sum_{km\in y} \left[ w_{ky}I\left( k\in r,y \right)I\left( e_{fkm}>0 \right) \right]=0$ for fruit *f* then $\hat{P}_{fmry}$ does not exist. This occurs when no household *k* that resides in market *r* in year *y* purchases *f* in month *m*.

We created another set of imputed prices with the following method. The observed price of fruit type $\times$ variety *f* in month *m* in in market *r* in year *y* is explained by the following model,

$E\left[ P_{fkmy} \right]=\alpha+\sum_{m=2}^{12} \beta_{m}I(m)+\sum_{r=2}^{76} \gamma_{r}I(r)+\sum_{f=2}^{20} \theta_{f}I(f)$ (E)

where *I*(*m*) is a dummy variable that equals 1 if the observed month is equal to *m* and equals 0 otherwise, *I*(*r*) is a dummy variable that equals 1 if the observed household *k* is in region *r* in year *y* and equals 0 otherwise, and *I*(*f*) is a dummy variable that equals 1 if the observed fruit is equal to *f* and equals 0 otherwise. The set of $\alpha$, $\boldsymbol{\beta}$, $\boldsymbol{\gamma}$, and $\boldsymbol{\theta}$ are model coefficients to be estimated. We estimate (Z) for each year in our dataset using weighted OLS where *k*’s projection factor in year *y* as the weight (i.e., we use the [pweight=projection factor] option after reg in Stata). Let the expected price of fruit *f* in month *m* in region *r* in year y be given by,

${\hat{\hat{P}}}_{fmry}=\hat{\alpha}+\hat{\beta}_{m}+\hat{\gamma}_{r}+\hat{\theta}_{f}$ (F)

where $\hat{\beta}_{m}=0$ if *m* = 1, $\hat{\gamma}_{r}=0$ if *r* = 1, and $\hat{\theta}_{f}=0$ if *f* = 1.

If *P_fkm_* does not exist then *P_fkm_* is set equal to the appropriate $\hat{P}_{fmry}$ (household-month *km* is assigned the $\hat{P}_{fmry}$ that matches *km*’s market of residence and the month of time). If the appropriate $\hat{P}_{fmry}$ does not exist then *P_fkm_* is set equal to the appropriate ${\hat{\hat{P}}}_{fmry}$.
