# Supplementary material for: The distributional impact of a green payment policy for organic fruit
Source: PLoS One. 2019 Feb 7;14(2):e0211199. doi: 10.1371/journal.pone.0211199 (PMC6366746; doi:10.1371/journal.pone.0211199)
Supplement: S3 Supporting information — (DOCX) [file pone.0211199.s018.docx]

**S3 Supporting information. Determining a household’s income class.**

A household *k* is poor if its annual income in *m*’s calendar year was 130% or less of the federal poverty line (FPL), conditional on year and household size. A household *k* is middle class if it annual income in *m*’s calendar year was 130% and 500% of the FPL, conditional on year and household size. A household *k* is rich if its annual income in *m*’s calendar year was greater than 500% of the FPL, conditional on year and household size. We defined a household at 130% of the FPL and below as poor as that defines the threshold for Supplemental Nutrition Assistance Program eligibility (see <https://www.fns.usda.gov/snap/eligibility>). We defined a household at 500% of the FPL and greater as rich because households are not eligible for many government and non-profit assistance programs at that level of income. For example, “[t]o be eligible for any of the Leukemia and Lymphoma financial assistance programs you must “have a household income that is at or below 500 percent of the U.S. federal poverty level guidelines as adjusted by the Cost of Living Index.” (<https://www.lls.org/sites/default/files/National/USA/Pdf/Slides_Transcipts/2018_FPL_English.pdf>). Further, many states cut off AIDS Drug Assistance Program eligibility at 500% of FPL (<https://www.kff.org/23a2da2/>)

In S3 Supporting Information Table 1 we show household FPL income (nominal), 130 percent of the FPL, and 500 percent of the FPL conditional on year and number of people in household.

**S3 Supporting Information Table 1**

|  | **Poverty Line** | | | **130% of poverty line** | | | **500% of poverty line** | | |
| --- | --- | --- | --- | --- | --- | --- | --- | --- | --- |
| **Persons in Family** | **2011** | **2012** | **2013** | **2011** | **2012** | **2013** | **2011** | **2012** | **2013** |
| 1 | 10,890 | 11,170 | 11,490 | 14,157 | 14,521 | 14,937 | 54,450 | 55,850 | 57,450 |
| 2 | 14,710 | 15,130 | 15,510 | 19,123 | 19,669 | 20,163 | 73,550 | 75,650 | 77,550 |
| 3 | 18,530 | 19,090 | 19,530 | 24,089 | 24,817 | 25,389 | 92,650 | 95,450 | 97,650 |
| 4 | 22,350 | 23,050 | 23,550 | 29,055 | 29,965 | 30,615 | 111,750 | 115,250 | 117,750 |
| 5 | 26,170 | 27,010 | 27,570 | 34,021 | 35,113 | 35,841 | 130,850 | 135,050 | 137,850 |
| 6 | 29,990 | 30,970 | 31,590 | 38,987 | 40,261 | 41,067 | 149,950 | 154,850 | 157,950 |
| 7 | 33,810 | 34,930 | 35,610 | 43,953 | 45,409 | 46,293 | 169,050 | 174,650 | 178,050 |
| 8 | 37,630 | 38,890 | 39,630 | 48,919 | 50,557 | 51,519 | 188,150 | 194,450 | 198,150 |

See <https://aspe.hhs.gov/2011-hhs-poverty-guidelines>; <https://aspe.hhs.gov/2012-hhs-poverty-guidelines>; and <https://aspe.hhs.gov/2013-poverty-guidelines> for information on poverty line conditional on year and household size.

Each household month *km* has an observed income category. In S3 Supporting Information Table 2 we indicate which income categories were assigned to which income class.

**S2 Supporting Information Table 2**

| **2011** | | | | | | **2012** | | | | | |
| --- | --- | --- | --- | --- | --- | --- | --- | --- | --- | --- | --- |
| **Poor** | | | **Rich** | | | **Poor** | | | **Rich** | | |
| **HH Inc Cat** | **Annual Income** | **HH Size** | **HH Inc Cat** | **Annual Income** | **HH Size** | **HH Inc Cat** | **Annual Income** | **HH Size** | **HH Inc Cat** | **Annual Income** | **HH Size** |
| 3 | 2500 | 1 | 21 | 55000 | 1 | 3 | 2500 | 1 | 21 | 55000 | 1 |
| 4 | 6500 | 1 | 23 | 65000 | 1 | 4 | 6500 | 1 | 23 | 65000 | 1 |
| 6 | 9000 | 1 | 26 | 80000 | 1 | 6 | 9000 | 1 | 26 | 80000 | 1 |
| 8 | 11000 | 1 | 27 | 150000 | 1 | 8 | 11000 | 1 | 27 | 150000 | 1 |
| 10 | 13500 | 1 | 26 | 80000 | 2 | 10 | 13500 | 1 | 26 | 80000 | 2 |
| 3 | 2500 | 2 | 27 | 150000 | 2 | 3 | 2500 | 2 | 27 | 150000 | 2 |
| 4 | 6500 | 2 | 26 | 80000 | 3 | 4 | 6500 | 2 | 26 | 80000 | 3 |
| 6 | 9000 | 2 | 27 | 150000 | 3 | 6 | 9000 | 2 | 27 | 150000 | 3 |
| 8 | 11000 | 2 | 27 | 150000 | 4 | 8 | 11000 | 2 | 27 | 150000 | 4 |
| 10 | 13500 | 2 | 27 | 150000 | 5 | 10 | 13500 | 2 | 27 | 150000 | 5 |
| 11 | 17500 | 2 | 27 | 150000 | 6 | 11 | 17500 | 2 | 27 | 150000 | 6 |
| 3 | 2500 | 3 | 27 | 150000 | 7 | 3 | 2500 | 3 | 27 | 150000 | 7 |
| 4 | 6500 | 3 | 27 | 150000 | 8 | 4 | 6500 | 3 | 27 | 150000 | 8 |
| 6 | 9000 | 3 |  |  |  | 6 | 9000 | 3 |  |  |  |
| 8 | 11000 | 3 |  |  |  | 8 | 11000 | 3 |  |  |  |
| 10 | 13500 | 3 |  |  |  | 10 | 13500 | 3 |  |  |  |
| 11 | 17500 | 3 |  |  |  | 11 | 17500 | 3 |  |  |  |
| 13 | 22500 | 3 |  |  |  | 13 | 22500 | 3 |  |  |  |
| 3 | 2500 | 4 |  |  |  | 3 | 2500 | 4 |  |  |  |
| 4 | 6500 | 4 |  |  |  | 4 | 6500 | 4 |  |  |  |
| 6 | 9000 | 4 |  |  |  | 6 | 9000 | 4 |  |  |  |
| 8 | 11000 | 4 |  |  |  | 8 | 11000 | 4 |  |  |  |
| 10 | 13500 | 4 |  |  |  | 10 | 13500 | 4 |  |  |  |
| 11 | 17500 | 4 |  |  |  | 11 | 17500 | 4 |  |  |  |
| 13 | 22500 | 4 |  |  |  | 13 | 22500 | 4 |  |  |  |
| 15 | 27500 | 4 |  |  |  | 15 | 27500 | 4 |  |  |  |
| 3 | 2500 | 5 |  |  |  | 3 | 2500 | 5 |  |  |  |
| 4 | 6500 | 5 |  |  |  | 4 | 6500 | 5 |  |  |  |
| 6 | 9000 | 5 |  |  |  | 6 | 9000 | 5 |  |  |  |
| 8 | 11000 | 5 |  |  |  | 8 | 11000 | 5 |  |  |  |
| 10 | 13500 | 5 |  |  |  | 10 | 13500 | 5 |  |  |  |
| 11 | 17500 | 5 |  |  |  | 11 | 17500 | 5 |  |  |  |
| 13 | 22500 | 5 |  |  |  | 13 | 22500 | 5 |  |  |  |
| 15 | 27500 | 5 |  |  |  | 15 | 27500 | 5 |  |  |  |
| 16 | 32500 | 5 |  |  |  | 16 | 32500 | 5 |  |  |  |
| 3 | 2500 | 6 |  |  |  | 3 | 2500 | 6 |  |  |  |
| 4 | 6500 | 6 |  |  |  | 4 | 6500 | 6 |  |  |  |
| 6 | 9000 | 6 |  |  |  | 6 | 9000 | 6 |  |  |  |
| 8 | 11000 | 6 |  |  |  | 8 | 11000 | 6 |  |  |  |
| 10 | 13500 | 6 |  |  |  | 10 | 13500 | 6 |  |  |  |
| 11 | 17500 | 6 |  |  |  | 11 | 17500 | 6 |  |  |  |
| 13 | 22500 | 6 |  |  |  | 13 | 22500 | 6 |  |  |  |
| 15 | 27500 | 6 |  |  |  | 15 | 27500 | 6 |  |  |  |
| 16 | 32500 | 6 |  |  |  | 16 | 32500 | 6 |  |  |  |
| 17 | 37500 | 6 |  |  |  | 17 | 37500 | 6 |  |  |  |
| 3 | 2500 | 7 |  |  |  | 3 | 2500 | 7 |  |  |  |
| 4 | 6500 | 7 |  |  |  | 4 | 6500 | 7 |  |  |  |
| 6 | 9000 | 7 |  |  |  | 6 | 9000 | 7 |  |  |  |
| 8 | 11000 | 7 |  |  |  | 8 | 11000 | 7 |  |  |  |
| 10 | 13500 | 7 |  |  |  | 10 | 13500 | 7 |  |  |  |
| 11 | 17500 | 7 |  |  |  | 11 | 17500 | 7 |  |  |  |
| 13 | 22500 | 7 |  |  |  | 13 | 22500 | 7 |  |  |  |
| 15 | 27500 | 7 |  |  |  | 15 | 27500 | 7 |  |  |  |
| 16 | 32500 | 7 |  |  |  | 16 | 32500 | 7 |  |  |  |
| 17 | 37500 | 7 |  |  |  | 17 | 37500 | 7 |  |  |  |
| 18 | 42500 | 7 |  |  |  | 18 | 42500 | 7 |  |  |  |
| 3 | 2500 | 8 |  |  |  | 3 | 2500 | 8 |  |  |  |
| 4 | 6500 | 8 |  |  |  | 4 | 6500 | 8 |  |  |  |
| 6 | 9000 | 8 |  |  |  | 6 | 9000 | 8 |  |  |  |
| 8 | 11000 | 8 |  |  |  | 8 | 11000 | 8 |  |  |  |
| 10 | 13500 | 8 |  |  |  | 10 | 13500 | 8 |  |  |  |
| 11 | 17500 | 8 |  |  |  | 11 | 17500 | 8 |  |  |  |
| 13 | 22500 | 8 |  |  |  | 13 | 22500 | 8 |  |  |  |
| 15 | 27500 | 8 |  |  |  | 15 | 27500 | 8 |  |  |  |
| 16 | 32500 | 8 |  |  |  | 16 | 32500 | 8 |  |  |  |
| 17 | 37500 | 8 |  |  |  | 17 | 37500 | 8 |  |  |  |
| 18 | 42500 | 8 |  |  |  | 18 | 42500 | 8 |  |  |  |
| 19 | 47500 | 8 |  |  |  | 19 | 47500 | 8 |  |  |  |

**S3 Supporting Information Table 2 (cont.)**

| **2013** | | | | | |
| --- | --- | --- | --- | --- | --- |
| **Poor** | | | **Rich** | | |
| **HH Inc Cat** | **Annual Income** | **HH Size** | **HH Inc Cat** | **Annual Income** | **HH Size** |
| 3 | 2500 | 3 | 21 | 55000 | 1 |
| 4 | 6500 | 4 | 23 | 65000 | 1 |
| 6 | 9000 | 6 | 26 | 80000 | 1 |
| 8 | 11000 | 8 | 27 | 150000 | 1 |
| 10 | 13500 | 10 | 26 | 80000 | 2 |
| 3 | 2500 | 3 | 27 | 150000 | 2 |
| 4 | 6500 | 4 | 26 | 80000 | 3 |
| 6 | 9000 | 6 | 27 | 150000 | 3 |
| 8 | 11000 | 8 | 27 | 150000 | 4 |
| 10 | 13500 | 10 | 27 | 150000 | 5 |
| 11 | 17500 | 11 | 27 | 150000 | 6 |
| 3 | 2500 | 3 | 27 | 150000 | 7 |
| 4 | 6500 | 4 | 27 | 150000 | 8 |
| 6 | 9000 | 6 |  |  |  |
| 8 | 11000 | 8 |  |  |  |
| 10 | 13500 | 10 |  |  |  |
| 11 | 17500 | 11 |  |  |  |
| 13 | 22500 | 13 |  |  |  |
| 3 | 2500 | 3 |  |  |  |
| 4 | 6500 | 4 |  |  |  |
| 6 | 9000 | 6 |  |  |  |
| 8 | 11000 | 8 |  |  |  |
| 10 | 13500 | 10 |  |  |  |
| 11 | 17500 | 11 |  |  |  |
| 13 | 22500 | 13 |  |  |  |
| 15 | 27500 | 15 |  |  |  |
| 3 | 2500 | 3 |  |  |  |
| 4 | 6500 | 4 |  |  |  |
| 6 | 9000 | 6 |  |  |  |
| 8 | 11000 | 8 |  |  |  |
| 10 | 13500 | 10 |  |  |  |
| 11 | 17500 | 11 |  |  |  |
| 13 | 22500 | 13 |  |  |  |
| 15 | 27500 | 15 |  |  |  |
| 16 | 32500 | 16 |  |  |  |
| 3 | 2500 | 3 |  |  |  |
| 4 | 6500 | 4 |  |  |  |
| 6 | 9000 | 6 |  |  |  |
| 8 | 11000 | 8 |  |  |  |
| 10 | 13500 | 10 |  |  |  |
| 11 | 17500 | 11 |  |  |  |
| 13 | 22500 | 13 |  |  |  |
| 15 | 27500 | 15 |  |  |  |
| 16 | 32500 | 16 |  |  |  |
| 17 | 37500 | 17 |  |  |  |
| 3 | 2500 | 3 |  |  |  |
| 4 | 6500 | 4 |  |  |  |
| 6 | 9000 | 6 |  |  |  |
| 8 | 11000 | 8 |  |  |  |
| 10 | 13500 | 10 |  |  |  |
| 11 | 17500 | 11 |  |  |  |
| 13 | 22500 | 13 |  |  |  |
| 15 | 27500 | 15 |  |  |  |
| 16 | 32500 | 16 |  |  |  |
| 17 | 37500 | 17 |  |  |  |
| 18 | 42500 | 18 |  |  |  |
| 3 | 2500 | 3 |  |  |  |
| 4 | 6500 | 4 |  |  |  |
| 6 | 9000 | 6 |  |  |  |
| 8 | 11000 | 8 |  |  |  |
| 10 | 13500 | 10 |  |  |  |
| 11 | 17500 | 11 |  |  |  |
| 13 | 22500 | 13 |  |  |  |
| 15 | 27500 | 15 |  |  |  |
| 16 | 32500 | 16 |  |  |  |
| 17 | 37500 | 17 |  |  |  |
| 18 | 42500 | 18 |  |  |  |
| 19 | 47500 | 19 |  |  |  |
