# Supplementary material for: The distributional impact of a green payment policy for organic fruit
Source: PLoS One. 2019 Feb 7;14(2):e0211199. doi: 10.1371/journal.pone.0211199 (PMC6366746; doi:10.1371/journal.pone.0211199)
Supplement: S4 Supporting information — (DOCX) [file pone.0211199.s019.docx]

**S4 Supporting information. Data manipulation.**

We reduce the 20 fruit type $\times$ varieties to 10 fruit type $\times$ varieties before we conduct numerical analysis. We do this by first combining *km*’s expenditures on and ounces purchased of blackberry $\times$ conventional; grape $\times$ conventional, grapefruit $\times$ conventional, lemon $\times$ conventional, raspberry $\times$ conventional, and other$\times$ conventional. Let this new fruit type $\times$ variety be known as other$\times$ conventional. Next we combine *km*’s expenditures on and ounces purchased of blackberry $\times$ organic; grape $\times$ organic, grapefruit $\times$ organic, lemon $\times$ organic, raspberry $\times$ organic, and other$\times$ organic. Let this new fruit type $\times$ variety be known as other$\times$ organic. Now we have ten $e_{ikm}$ and $o_{ikm}$variables where *i* indexes the set of 10 fruit type $\times$ varieties.

To calculate *P_ikm_* for *i =* other$\times$ organic and *i =* other$\times$ conventional for household *k* in month *m* we do the following. First let *u* index the 6 organic fruit types that we collapsed into one category. Define $\bar{e}_{ukm\in y}$as,

$\bar{e}_{uy}=\frac{1}{N}\sum_{km\in y}^{N} e_{ukm}$ (A)

$\bar{e}_{Ukm\in y}$as,

$\bar{e}_{Uy}=\frac{1}{N}\left( \sum_{u=1}^{6} \sum_{km\in y}^{N} e_{ukm} \right)$ (B)

and,

${share}_{uy}=\bar{e}_{uy}/\bar{e}_{Uy}$ (C)

Finally,

$P_{ikm}=\sum_{u=1}^{6} share_{uy}P_{ukm\in y}$ (D)

for *i =* other$\times$ organic. To calculate *P_ikm_* for *i =* other$\times$ conventional we repeat (A) - (D) using the appropriate expenditures and prices.

In the Nielsen data annual household income is coded in categories. We recode income categories using the following where the number before the equal sign is the category and the number after the equal sign is the nominal dollar amount we assumed,

(3=2500); (4=6500); (6=9000); (8=11000); (10=13500); (11=17500); (13=22500); (15=27500); (16=32500); (17=37500); (18=42500); (19=47500); (21=55000); (23=65000); (26=80000); (27=150000)

We convert annual household income to monthly real household income by dividing by 12 and then inflating according to the CPI.
