# Supplementary material for: The distributional impact of a green payment policy for organic fruit
Source: PLoS One. 2019 Feb 7;14(2):e0211199. doi: 10.1371/journal.pone.0211199 (PMC6366746; doi:10.1371/journal.pone.0211199)
Supplement: S5 Supporting information — (DOCX) [file pone.0211199.s020.docx]

**S5 Supporting information. Organic fruit demand estimation methods.**

*Separate equations method (estimation method 1)*

In the file Stata .do file ‘HeckmanConditionalElasticities.do’ we provide code to estimate the Heckman conditional elasticities and related conditional demand values found in the in the ‘Heckman’ rows of Table 4. The code uses the .dta file ‘AllExp’. The code is specifically designed to estimate conditional demand for organic strawberries at the representative rich household. Instructions in the code explain how it can be changed to generate results for other fruits and/or household types.

In the file Stata .do file ‘HeckmanUnconditionalElasticities.do’ we provide code to estimate the Heckman unconditional elasticities and related unconditional demand values found in the in the ‘Heckman’ rows of Table 5. The code uses the .dta file ‘AllExp’. The code is specifically designed to estimate unconditional quantity demanded of organic strawberries at the representative rich household. Instructions in the code explain how it can be changed to generate results for other fruits and/or household types.

Please note that the OLS estimator of the fruit consumed equation (i.e., $E\left[o_{ikm}|e_{ikm}>0\right]$) does not treat a household-month's predicted inverse mills ratio as the random variable it is. Instead the estimator treats it as a scalar. Therefore, the estimated standard errors of the OLS-derived coefficients will be smaller than the true standard errors. Because the standard errors of OLS-estimated coefficients are biased the standard errors of predicted infra-marginal savings, change in purchases, and elasticities will be smaller than their true standard errors as well.

Finally, all predicted conditional and unconditional elasticities and other related demand values assume the representative household-month is from the Boston market (the omitted market in the selection equation). We assumed this when demand was predicted with estimation method 1 because Stata’s nlcom function, a vital function in estimation method 1’s code, has a limit on how many independent variables can be used. However, this assumption has a negligible effect on predictions. Estimation method 2 also assumed the representative households were from Boston. In contrast, estimation method 3 (the LASSO method) used all markets when predicting conditional and unconditional elasticities and other related demand values.

The files ‘AllExp.dta’, ‘HeckmanUnconditionalElasticities.do’, and ‘HeckmanConditionalElasticities.do’ are found in the zip file ‘SeparateEqPredictions(Method1)’ at an OpenICPSR project with the persistent URL: <http://doi.org/10.3886/E108101V2>.

*LinQuad method (estimation method 2)*

Here we describe the data and code provided to estimate the LinQuad unconditional and conditional elasticities and related demand values found in the in the ‘LinQuad’ rows of Tables 4 and 5. All of the code uses the .dta file ‘AllExp’.

The Stata .do files ‘linquad_8_X_allexp_v4a1.do’ estimate each X’s unconditional and conditional organic and conventional fruit demand system and saves the coefficients where X={poor, middle, or rich household-month}. As in estimation method 1, $\hat{\lambda}_{ikm}$, , and $\Phi\left(\mathbf{X}_{km}\hat{\delta}_{i}+\mathbf{C}_{km}\hat{\mu}_{i}+\mathbf{P}_{km}\hat{\omega}_{i}\right)$ are predicted one fruit at a time for each *km* $\in$*z* using a first stage probit. The selection demand equations potentially could be estimated as a system. However, we will follow Shonkwiler and Yen (1999) and use equation by equation treatment of selection for positive quantities. Equation by equation treatment of the positive selection, although consistent, ignores useful cross equation information about selection. Yen and Lin (2002) propose a method with correlated selection terms for a linear demand system and Yen et al. (2003) use correlated selection terms in a nonlinear Translog demand system. Further, as we note in the text, following the LinQuad method, we use a seemingly unrelated regression (SUR) approach to estimate$f\left(\mathbf{X}_{km}\beta_{i},\mathbf{c}_{km}\sigma_{i},\mathbf{P}_{km}\theta_{i}\right)$’s parameters $\beta,\sigma,\alpha,\theta,$ and $\psi$across all *i* jointly (less organic-other and conventional-other) using *all* household-months *km* $\in$*z* with imposed symmetry in the price coefficients and homogeneity in prices and income. Symmetry in price coefficients means that θ*_ij_* = θ*_ji_*,for all *i*,*j* combinations where j also indexes fruit type $\times$ variety. Homogeneity in prices and income is imposed by deflating all prices and income by a common deflator. Technically, the deflator should be an index of all prices except those for fruit. We deflated by the CPI-U and assumed that the CPI’s inclusion of fruit prices would be inconsequential.

The Stata .do files ‘linquad_8sim_X_allexp_v4b1.do’ simulate the impact of a 10% subsidy on all organic fruits on X’s unconditional and conditional organic and conventional fruit demand system where X={poor, middle, or rich household-month}. As in estimation method 1, all predicted conditional and unconditional elasticities and other related demand values assume the representative household-month is from the Boston market (the omitted market in the selection equation). In contrast, estimation method 3 (the LASSO method) used all markets when predicting conditional and unconditional elasticities and other related demand values.

The Stata .do files ‘linquad_8sim_X_allexp_v4b1_stnderr.do’ generates standard errors for all values reported in Tables 5 and 6. Finally, the Stata .do files ‘linquad_8sim_X_allexp_v4b1_getmeans.do’ generates household-month means for each X where X={poor, middle, or rich household-month}. The means vary by income class and fruit when predicting conditional demand elasticities and related conditional demand values.

The seven Stata .do files associated with the LinQuad estimates are found in the zip file ‘LinQuadPredictions(Method2)’ at <https://www.openicpsr.org/openicpsr/project/108101/version/V1/view>. The data file ‘AllExp.dta’ found in the zip file ‘SeparateEqPredictions(Method1)’ at an OpenICPSR project with the persistent URL: <http://doi.org/10.3886/E108101V2>.

*LASSO method (estimation method 3)*

In the R file ‘organic fruit demand 19 submitted annotated.R’ we provide code to estimate the LASSO conditional and unconditional elasticities and other related demand model statistics found in the LASSO rows of Tables 4 and 5. The code uses the .csv files ‘AllExp2011.csv’, ‘AllExp2012.csv’, and ‘AllExp2013.csv’. The code is specifically designed to estimate demand for the pooled years sample, iterating across fruits and income categories, with 100 bootstrap replicates for each model. Instructions in the code explain how it can be changed to generate results for other combinations of years, fruits, and income categories. The data files and the R file associated with the LASSO estimates are found in the zip file ‘LASSOPredictions(Method3)’ at an OpenICPSR project with the persistent URL: <http://doi.org/10.3886/E108101V2>.

The script consists of initialization code, function definitions, and, lastly, the loop across models and bootstrap procedure. The following describes the initialization, definitions, and then details a complete example iteration:

1. The environment is cleared and parallelization of threads is initialized (available for only some packages and computers).
2. The “response.sim” function is defined. This function runs simulations using an estimated model of demand and two samples: the original sample and a simulation (counterfactual) sample. For example, a price elasticity can be calculated by raising the price in the simulation sample, then calculating the average change in demand, and finally by dividing the proportional changes. The function returns all statistics of interest, including quantity changes and elasticities. It is described in more detail below.
3. The “demand.lasso” function is defined. This function estimates the LASSO demand model. It is described in more detail below.
4. The “dataprep” function is defined. This function prepares the raw data for estimating the demand model.
5. After all of the functions have been defined (at the end of the file), the “for” loop iterates across fruits and household types (these iterations can be changed using the various commented versions in the code). For each iteration, the “boot” package is used to obtain bootstrapped estimates of the standard errors of elasticities, etc, using the functions defined above. First, the “dataprep” function is called, then the “demand.lasso” function (which itself calls the “response.sim” function).
   1. The “dataprep” function prepares the estimation sample by:
      1. Combining the specified year files.
      2. Performing a series of additional data cleaning commands.
      3. Creating training and test samples for model evaluation (LASSO models are trained on a random 80% sample).
      4. Selecting a subset for a particular income category, if specified.
      5. Creating conventional & organic, expenditure, price, and quantity matrices.
      6. Creating continuous and dummy dependent variables for estimation.
      7. The function returns the “us” sample, which is the unconditional sample to be used in estimation and elasticity simulations.
   2. With the “us” sample defined, the “demand.lasso” function next estimates the LASSO models.
      1. Define the statistics to be simulated after the LASSO estimation.
      2. Estimate the unconditional probability of any purchase using a logistic regression LASSO model (binomial family), with the dependent variable as a dummy indicating any purchase.
      3. For each observation, predict the probability of any purchase using the trained model (propensity score to be used below).
      4. Evaluate model performance using the held out test sample (ROC curve and area under the curve).
      5. Using the “conditional sample”, restricted to those observations with a positive purchase quantity, estimate a linear LASSO model predicting quantity purchased. Use all predictor variables, including propensity score from the first stage.
      6. For each observation, predict the quantity purchased using the trained model (the predicted value from the linear LASSO model estimated in step v, times the predicted probability of purchase obtained in step iii).
      7. Evaluate model performance using the held out test sample.
   3. At the end of the “demand.lasso” function, the “response.sim” function is called in order to calculate elasticities, etc. The simulations proceed by calculating the mean predicted quantity for all observations in the original sample and comparing that mean against the mean predicted quantity in the sample with a modified independent variable, e.g. price change. The following simulations are run:
      1. 10% price increase for each fruit price (conventional and organic), to estimate own- and cross-price elasticities.
      2. 10% increase in income to estimate income elasticity.
      3. 10% subsidy on all organic fruits.
      4. 10% tax on all conventional fruits.
      5. When called, the “response.sim” function performs the following.
      6. Calculate the simulated probability (propensity score) using the logistic regression LASSO model.
      7. Calculate the mean change in probability and the corresponding elasticity.
      8. Simulate changes in the second stage (quantity), after simulating the change in probability in the first stage. We calculate the predicted values for all observations, then calculate the conditional price elasticity by restricting attention to positive values and unconditional price elasticity by again using the full sample.
      9. Return all calculated estimates and elasticities of interest.

Shonkwiler JJS, Yen ST. Two-step Estimation of a Censored System of Equations. Am J Agr Econ. 1999; 81: 972-982.

Yen ST, Lin BH. Beverage consumption among US Children and Adolescents: full-information and quasi maximum likelihood estimation of a censored system. Eur Rev Agric Econ. 2002; 29(1): 85-103.

Yen ST, Lin BH, Smallwood DM. Quasi- and simulated-likelihood approaches to censored demand systems: food consumption by food stamp recipients in the United States. Am J Agr Econ. 2003; 85: 458–478.
